# Spreading of TDP-43 pathology via pyramidal tract induces ALS-like phenotypes in TDP-43 transgenic mice

**DOI:** 10.1186/s40478-020-01112-3

**Published:** 2021-01-18

**Authors:** Xuebing Ding, Zhi Xiang, Chi Qin, Yongkang Chen, Haiyan Tian, Lin Meng, Danhao Xia, Han Liu, Jia Song, Jun Fu, Mingming Ma, Xuejing Wang

**Affiliations:** 1 Department of Neurology, The First Affiliated Hospital of Zhengzhou University, Zhengzhou, 450052 Henan China; 2 Institute of Parkinson and Movement Disorder, Zhengzhou University, Zhengzhou, 450052 Henan China; 3 Department of Neurology, Affiliated People’s Hospital of Zhengzhou University, Henan Provincial People’s Hospital, Zhengzhou, 450003 Henan China

**Keywords:** Amyotrophic lateral sclerosis, Transactive response DNA-binding protein 43 kDa, Pyramidal tract, Preformed fibrils, Prion-like transmission

## Abstract

Transactive response DNA-binding protein 43 kDa (TDP-43) has been identified as the major component of ubiquitinated inclusions found in patients with sporadic amyotrophic lateral sclerosis (ALS). Increasing evidence suggests prion-like transmission of TDP-43 aggregates via neuroanatomic connection in vitro and pyramidal tract in vivo. However, it is still unknown whether the spreading of pathological TDP-43 sequentially via pyramidal tract can initiate ALS-like pathology and phenotypes. In this study, we reported that injection of TDP-43 preformed fibrils (PFFs) into the primary motor cortex (M1) of Thy1-e (IRES-TARDBP) 1 mice induced the spreading of pathological TDP-43 along pyramidal tract axons anterogradely. Moreover, TDP-43 PFFs-injected Thy1-e (IRES-TARDBP) 1 mice displayed ALS-like neuropathological features and symptoms, including motor dysfunctions and electrophysiological abnormalities. These findings provide direct evidence that transmission of pathological TDP-43 along pyramidal tract induces ALS-like phenotypes, which further suggest the potential mechanism for TDP-43 proteinopathy.

**Electronic supplementary material:**

The online version of this article (10.1186/s40478-020-01112-3) contains supplementary material, which is available to authorized users.

## Introduction

Amyotrophic lateral sclerosis (ALS) is a fatal progressive degenerative motor neuron disorder with unknown etiology [4, 37, 41]. Cytoplasmic transactive response DNA-binding protein 43 kDa (TDP-43) inclusion was identified as a pathological hallmark of patients with ALS [2, 25, 33]. Under physiological conditions, TDP-43 resides predominantly in the nucleus, and participates in regulating RNA metabolism, including translation, alternative splicing and transport [2, 3, 9, 33]. However, TDP-43 translocates to the cytoplasm and forms hyperphosphorylated, insoluble and ubiquitin-positive aggregates in ALS [2, 39]. Postmortem histological studies have identified stereotypical spreading patterns of phosphorylated TDP-43 (pTDP-43) pathology, and these findings suggest that pTDP-43 positive inclusions may spread along axons in the central nervous system [6, 7].

Accumulating clinical and experimental evidence suggests that ALS displays considerable properties similar to the prion diseases, with misfolded TDP-43 acting as a “prion-like” protein [39]. In biochemical studies, TDP-43 has been identified as an intrinsically aggregation-prone protein whose C-terminal domain is critical for spontaneous aggregation [13, 39]. Studies performed in vitro have demonstrated that pathological TDP-43 could transmit from cell to cell along axon in a self-templating manner [13, 34, 38]. Unfortunately, experimental evidence in vivo is still largely lacking. In 2018, Svahn and his colleagues first demonstrated the nucleo-cytoplasmic transport of TDP-43 using a zebrafish model [40]. Until recently, Porta et al. [36] showed that intracerebral injection of pathological TDP-43 derived from frontotemporal lobar degeneration with TDP-43 pathology (FTLD-TDP) brains led to the formation and trans-neuronal spreading of TDP-43 pathology in both CamKIIa-hTDP43NLSm and non-transgenic mice. Up to now, no animal model has been established to test whether the spreading of TDP-43 pathology could induce the pathological changes and clinical phenotypes of ALS.

Here, we generated a transgenic mouse line expressing human TDP-43 (hTDP-43) under the Thy1 promoter. Then, TDP-43 preformed fibrils (TDP-43 PFFs) were injected into the left caudal part of primary motor cortex (M1) region (M1-C) of Thy1-e (IRES-TARDBP) 1 mice (TDP-43 PFFs M1-C mice) or the left lateral part of M1 region (M1-L) of Thy1-e (IRES-TARDBP) 1 mice (TDP-43 PFFs M1-L mice), respectively [45]. As early as 1 month post-injection (mpi), numerous neurons near the injection site showed cytoplasmic pTDP-43 mislocation in both TDP-43 PFFs M1-C mice and TDP-43 PFFs M1-L mice. At 6 mpi, pTDP-43 pathology invaded into bilateral motor cortex, pyramidal tract, lower motor neuron (LMN), and corresponding motor regulatory nucleus in the two groups of TDP-43 PFFs-injected mice. Parallelly, TDP-43 PFFs-injected mice developed ALS-like syndromes, including motor dysfunction, electrophysiological abnormalities, learning and memory deficits in TDP-43 PFFs M1-C mice, and swallowing disorder in TDP-43 PFFs M1-L mice in a time-dependent manner. Taken together, we have established a novel mouse model of ALS and demonstrated that the spreading of pathological TDP-43 via axonal trajectories and synaptic contacts along pyramidal tract may contribute to the pathogenesis of ALS.

## Results

### Anterograde transsynaptic spread of pathological TDP-43 along pyramidal tract after intra-primary motor cortex injection of TDP-43 PFFs

**Fig. 1:**
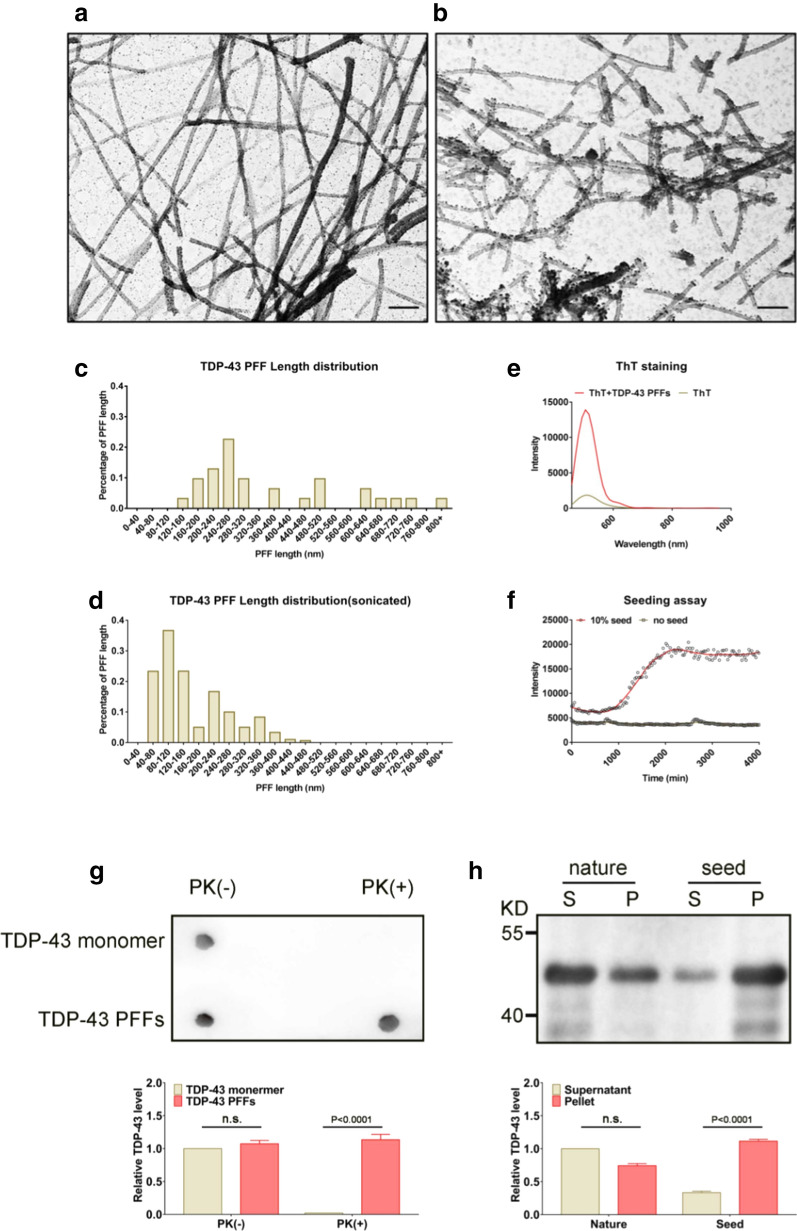
Structural and seeding property analysis of TDP-43 PFFs. Transmission electron microscope (TEM) images of TDP-43 PFFs before sonication (**a**) and after sonication (**b**). The statistics of the length of TDP-43 PFFs ranging from 120 nm to 800 nm before sonication (**c**) and 40 nm to 480 nm after sonication (**d**). n = 10 samples/group. Bar, 100 nm. Characteristic increase in ThT fluorescence upon binding to TDP-43 PFFs (**e**). A seeded fibrillation of TDP-43 fibrils monitored by the ThT fluorescence intensity (**f**). n = 10 samples/group. The fibril nature of the TDP-43 PFFs was confirmed by analysis of proteinase K (PK) resistance using the dot-blot (**g**). An SDS-PAGE analysis has shown that a higher level of insoluble TDP-43 was detected in the pellet fraction, but less TDP-43 remained in the soluble fraction after a seeding reaction (**h**).

We first examined the structure and morphology of TDP-43 PFFs using a transmission electron microscope (TEM), fluorescence spectroscopy with thioflavin T (ThT), and proteinase K (PK) resistance analysis. TEM analysis showed fibrillar protein with varying sizes, ranging from 120 to 800 nm length before sonication (Fig. 1a, c), and 40 to 480 nm after sonication (Fig. 1b, d). ThT fluorescence intensity was significantly increased after 5 min of phophate buffer incubation (Fig. 1e). Regarding TDP-43 PFFs seeding assay, the addition of 10% amounts of TDP-43 PFFs successfully triggered the fibrillation of soluble TDP-43 monomers. Without any agitation, TDP-43 monomers exhibited no increase in the intensity of ThT fluorescence and remained soluble for at least 3 days with no fibrillar aggregation. In contrast, even in the absence of agitation, the addition of 10% amounts of TDP-43 PFFs was sufficient to trigger the fibrillation of soluble TDP-43 monomers, which was confirmed by the increase in the ThT fluorescence intensity (Fig. 1f). PK resistance of TDP-43 PFFs was analyzed by the dot-blot test. The urea-fraction of TDP-43 solution dropped to significantly lower levels after agitation for 7 days (Fig. 1g). Furthermore, after a seeding reaction, a higher level of insoluble TDP-43 was detected in the pellet fraction, but less TDP-43 remained in the soluble fraction (Fig. 1h). These findings indicated the seeded fibrillation of TDP-43 PFFs in vitro.

To test whether the spreading of pathological TDP-43 via pyramidal tract can induce ALS-like phenotypes, we first generated a Thy1-e (IRES-TARDBP) 1 mouse line, specifically expressing hTDP-43 in nervous system, and evaluated the pathological phenotype of this mouse model on the behavior, molecular, and cellular levels. We detected hTDP-43 in M1, red nucleus (RN), inferior olive nucleus (ION), and cervical spinal cord anterior horn (Cs) of Thy1-e (IRES-TARDBP) 1 mice, and hTDP-43 was almost exclusively localized in the nucleus of neurons (Additional file 1: Fig. S1a-l). The expression level of hTDP-43 in Thy1-e (IRES-TARDBP) 1 mice were around 1.5-fold compared to the endogenous mouse TDP-43 (mTDP-43) in the cortex, and more than 2-fold in the spinal cord (Additional file 1: Fig. S1m-o). Moreover, pTDP-43-ir pathology was undetectable in PBS-injected Thy1-e (IRES-TARDBP) 1 mice at 8 mpi (Additional file 1: Fig. S2). Even at 20 mpi, no differences were found in rotarod test, hanging wire test, footprint test, Morris water maze test, weight, food and water consumption between Thy1-e (IRES-TARDBP) 1 mice and C57BL/6J mice (Additional file 1: Fig. S3).

**Fig. 2:**
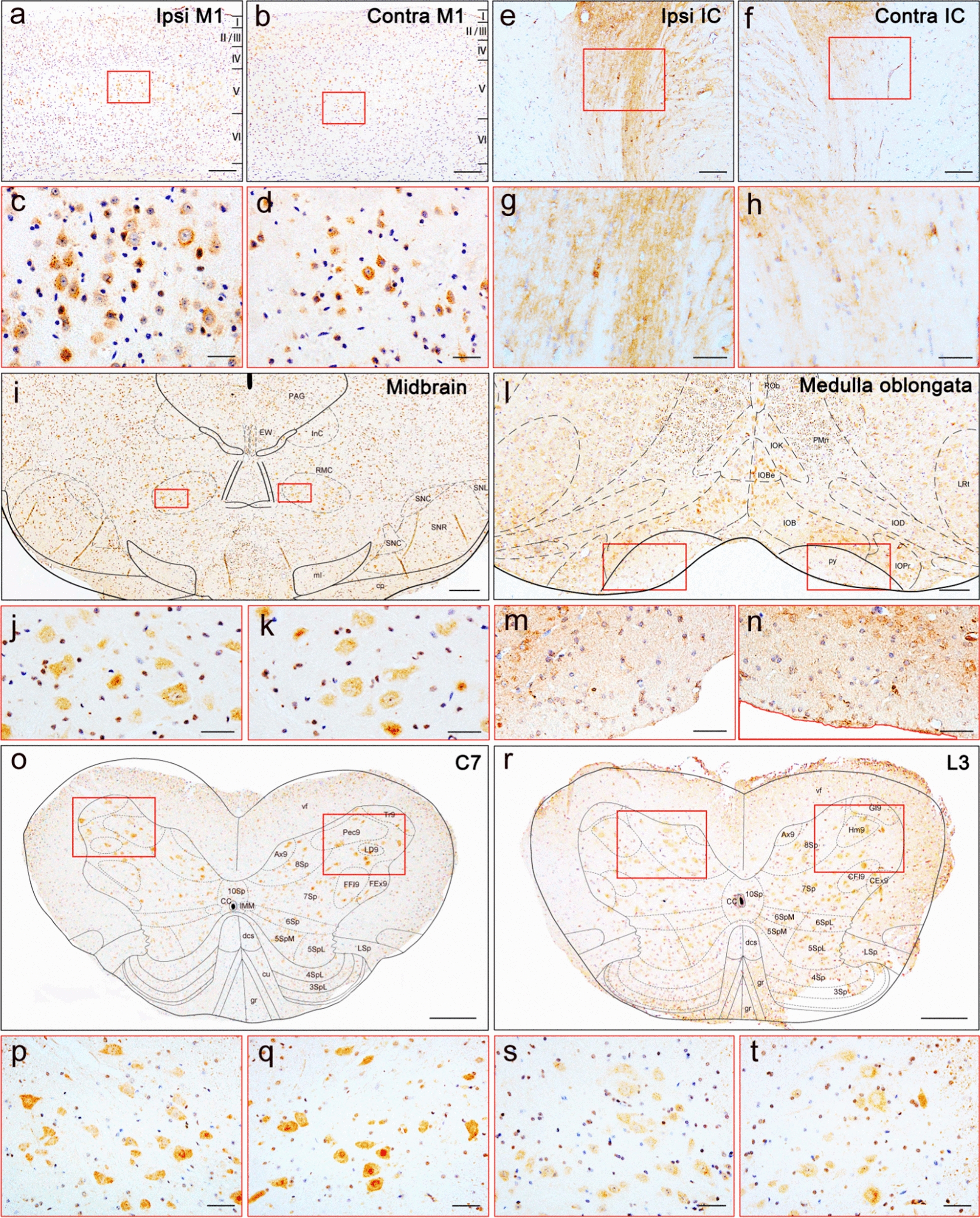
TDP-43 pathology distribution in different regions in TDP-43 PFFs-injected mice. Representative photomicrographs of p409-410 IHC staining in coronal brain sections of cortex (**a–d**), internal capsule (IC, **e–h**), midbrain (**i–k**), medulla oblongata (**l–n**), The seventh cervical spinal cord (C7, **o–q**), The third lumber spinal cord (L3, **r–t**) from TDP-43 M1-C mice analyzed at 8 mpi (n = 3 mice/group) in the ipsilateral (Ipsi) and contralateral (Contra) side of injection. In the cortex, p409–410 staining was mainly detected in the fifth layer of the ipsilateral primary motor cortex (M1, **a**, **c**) and contralateral M1 (**b**, **d**). **e–h** Show p409–410 staining in the Ipsi IC (**e**, **g**) and Contra IC (**f**, **h**). In the midbrain, **i–k** show p409-410 staining in the Ipsila red nucleus (RN, **j**), Contra RN (**k**) and bilateral substantia nigra (SN). In the medulla oblongata, **l–n** show p409–410 staining in the Ipsi decussatio pyramidum (py, **m**), Contra py (**n**) and inferior olive nucleus (ION). **o–q** show p409–410 staining in the Ipsi anterior horn of C7 (**p**) and Contra anterior horn of C7 (**q**). **r–t** show p409–410 staining in the Ipsila anterior horn of L3 (**s**) and Contra anterior horn of L3 (**t**). [Scale bars, a–b, 200 µm; c–d, p–q, s–t, j–k 50 µm; e–f, 100 µm; g–h, m–n, 20 µm; i, l, o, r, 500 µm]

**Fig. 3:**
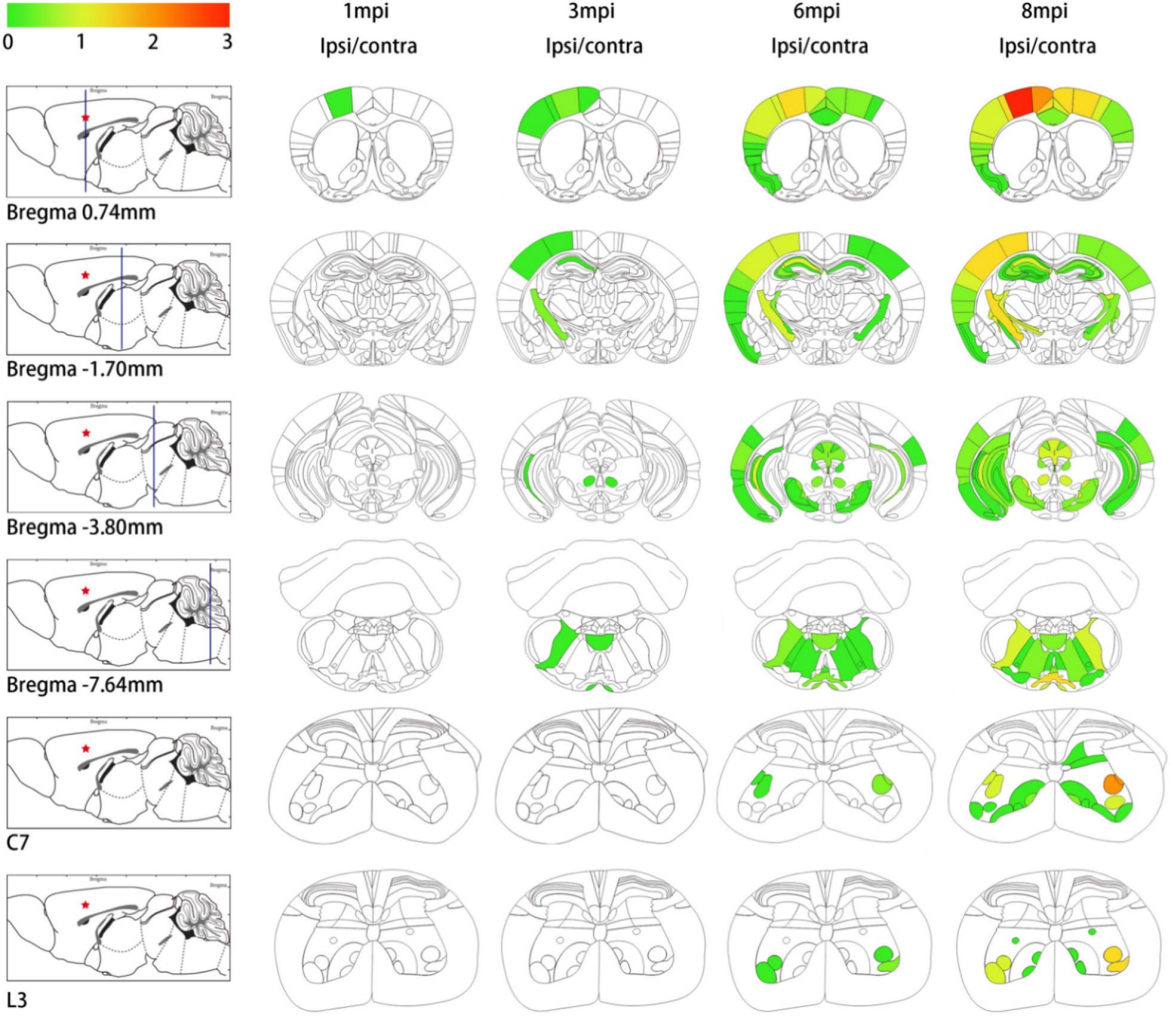
Heat maps of TDP-43 pathology in coronal sections stained with pTDP-43 antibody. Semi-quantitative analyses of the burden of TDP-43 pathology in neurons and in white matter tracts in the brain and spinal cord of TDP-43 M1-C mice at different times (n = 3 at 1 mpi, n = 3 at 2 mpi, n = 3 at 6 mpi, n = 3 at 12 mpi) after TDP-43 PFFs injection. Each panel represents heat map pathology distribution on one of the six different coronal planes (Bregma 0.74 mm, − 1.70 mm, − 3.80 mm, − 7.64 mm, C7 section, L3 section) for the different post-injection times. The mean values of the TDP-43 pathology were graded from negative (0, gray) to most abundant (3, red) and color-coded onto heat maps. The panels in the far left column shows sagittal views of the corresponding coronal planes (blue line) and the site of injection into cortex was indicated by the red star.

To determine whether inoculation of TDP-43 PFFs in M1 region could induce transmission of TDP-43 pathology along pyramidal tract, we injected TDP-43 PFFs into two different parts of left M1 [45]: M1-C, representing forelimb and hindlimb movements; and M1-L, representing tongue and jaw movements in Thy1-e (IRES-TARDBP) 1 mice (Additional file 1: Fig. S4). The mice were sacrificed at 1, 3, 6, 8, and 12 mpi. The brain tissues were subjected to immunohistochemistry (IHC), immunofluorescence (IF), and western blot (WB) to detect pathological TDP-43, total TDP-43 level and neuron loss. pTDP-43-immunoreactive (pTDP-43-ir) pathology was detected using an anti-pTDP-43 (phosphorylated at Ser409/Ser410) antibody at various time points. As early as 1 mpi, pTDP-43-ir pathology mainly distributed in the ipsilateral cortex around the injection regions in TDP-43 PFFs M1-C and M1-L mice, while pTDP-43-ir staining was not observed in the contralateral side. At 3 mpi, sparse cytoplasmic pTDP-43-ir staining was detected in the ipsilateral M1, secondary motor cortex (M2), primary somatosensory cortex (S1), field CA1 of hippocampus (CA1), internal capsule (IC), medullary reticular formation (MdRt), bilateral RN, hypoglossal nucleus (12N), and decussatio pyramidum (py) in TDP-43 PFFs M1-C mice. Simultaneously, pTDP-43-ir pathology was detected in the ipsilateral M1, IC, MdRt and bilateral 12N in TDP-43 PFFs M1-L mice. Over time, pTDP-43 pathology became much more extensive and possibly saturated. At 8 mpi, pTDP-43-ir staining was most abundant in bilateral M1, M2, S1, secondary somatosensory cortex (S2), cingulum (cg), CA1, field CA2 of hippocampus (CA2), field CA3 of hippocampus (CA3), pyramidal cell layer of the hippocampus, molecular layer of the dentate gyrus (Mol), IC, lateral globus pallidus (LGP), subthalamic nucleus (STh), reticular thalamic nucleus (Rt), stria terminalis (st), dorsomedial periaqueductal gray, interstitial nucleus of Cajal, substantia nigra (SN), medial lemniscus, cerebral peduncle, secondary visual cortex, temporal association cortex, MdRt, ION, paramedian reticular nucleus, RN, 12N, py, Cs, lumber spinal cord anterior horn (Ls) and ipsilateral agranular insular cortex, granular insular cortex, dysgranular insular cortex, piriform cortex, ectorhinal cortex, auditory cortex, perirhinal cortex, oriens layer of the hippocampus, dentate gyrus, zona incerta, optic tract, and lateral entorhinal cortex in TDP-43 PFFs M1-C mice (Fig. 2a–t). In addition, more severe pTDP-43 pathology was detected in ipsilateral brain regions to the injection site in M1, M2, S1, S2, cg, CA1, CA2, CA3, Mol, IC, LGP, Rt, st, and STh. However, in the contralateral regions, such as Cs and Ls, pTDP-43-ir pathological deposition became more severe than that in ipsilateral regions. In TDP-43 PFFs M1-L mice, pTDP-43 pathology became more abundant in bilateral 12N (Additional file 1: Fig. S5a-c). To further illustrate the spreading of TDP-43 pathology over time in TDP-43 PFFs M1-C mice, we produced a heat map to visualize the spatial distribution of pTDP-43 at different time points, as shown in Fig. 3. Additionally, the pTDP-43-ir staining was not detected in the brain of TDP-43 PFFs M1-injected C57BL/6J mice even at 20 mpi (Additional file 1: Fig. S6).

**Fig. 4:**
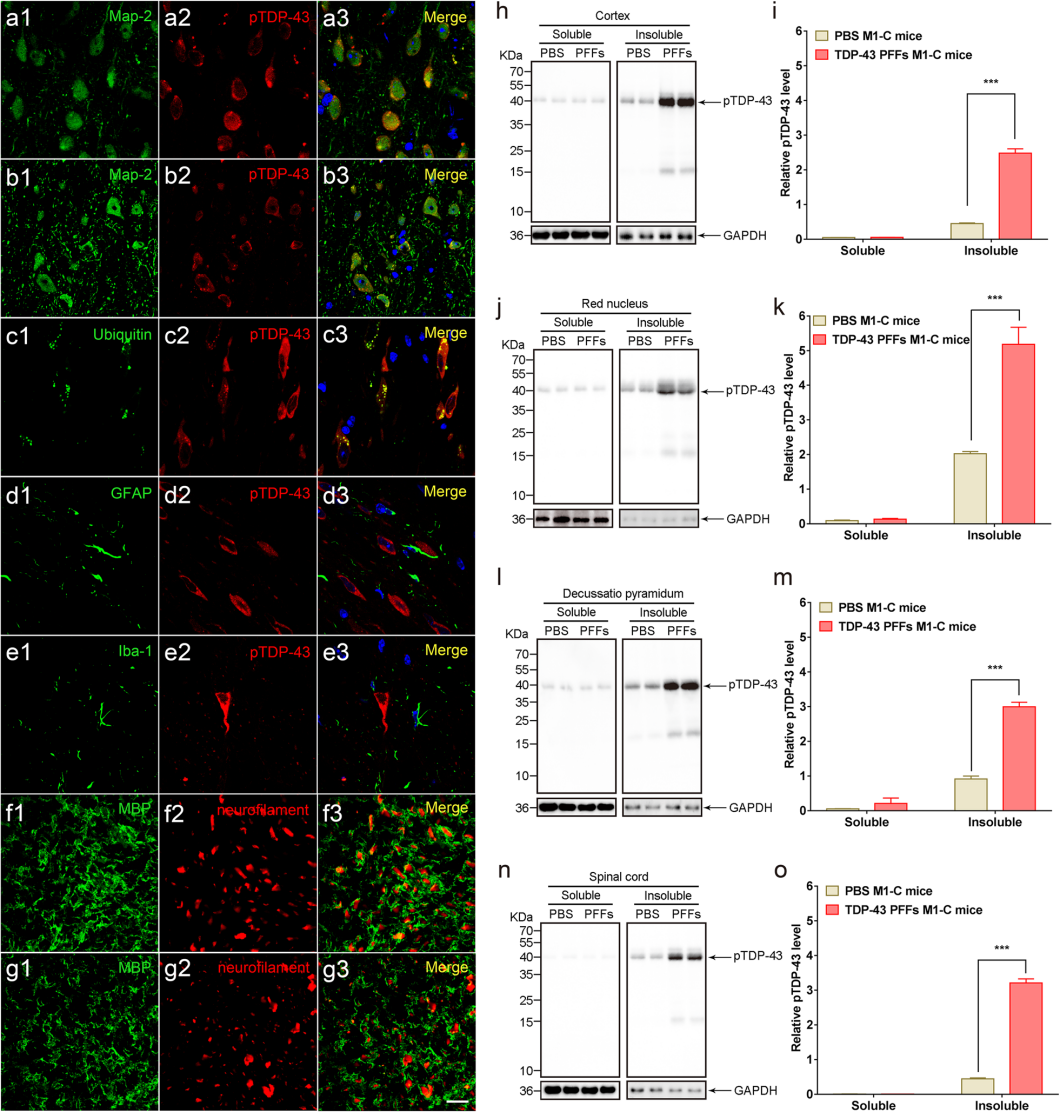
Double immunofluorescence analysis and Western blot analysis of TDP-43 PFFs M1-C mice at 4 mpi. **a1–e3** Representative images of immunofluorescence were analyzed to demonstrate the aggregation of pathological TDP-43 in different types of cells. Double immunofluorescence analysis of cortex, pons, medulla oblongata and cervical spinal cord from TDP-43 PFFs M1-C mice for pTDP-43 (red, **a2-e2**) with MAP2 in M1(green, **a1**) and parvicellular reticular nucleus (green, **b1**), ubiquitin in cervical spinal cord (green, **c1**), GFAP in LGP (green, **d1**), Iba-1 in MdD (green, **e1**). **f1–g3** Double immunolabeling for MBP (green) and neurofilament (red) in dorsal corticospinal tract in the ipsilateral side (**f1–f3**) and contralateral side (**g1–g3**) of injection from TDP-43 PFFs M1-C mice. Co-immunolabeling is represented by signal in yellow. Cell nuclei were counterstained with Hoechst 33258 (blue). [Scale bars, 30 μm (**a1–g3**)]. WB analysis of pTDP-43 in the soluble and insoluble fractions from cortex (**h**), red nucleus (**j**), decussatio pyramidum (**l**) and cervical spinal cord anterior horn (**n**) using the anti-pTDP-43 (phosphorylated at Ser409/Ser410) antibody. Blots were probed for GAPDH as a loading control (Bottom). Molecular weight markers of migrated protein standards are expressed in KDa. Quantification of soluble and insoluble pTDP-43 levels in cortex (**i**), red nucleus (**k**), decussatio pyramidum (**m**) and cervical spinal cord anterior horn (**o**) (n = 3 mice/group) show plentiful pTDP-43 in the extracts of TDP-43 PFFs M1-C mice while few in age-matched PBS M1-C mice. The error bar in panels (**i**, **k**, **m**, **o**) represents the Standard Error of Mean (SEM). Data are the mean ± SEM. Statistical significance was analyzed using the Student’s t test and Mann–Whitney test, ***P < 0.001.

To investigate the deposition of pathological TDP-43 in different types of cells in the brain and spinal cord, we performed a series of double IF staining with pTDP-43 antibodies and specific cellular marker antibodies in different brain regions of TDP-43 PFFs M1-C mice at 4 mpi. We used microtubule-associated protein-2 (MAP-2) for neurons, glial fibrillary acidic protein (GFAP) for astrocytes, ionized calcium binding adapter molecule 1 (Iba-1) for microglia, myelin basic protein (MBP) for oligodendrocytes, and ubiquitin (Ub) for aggregates. Double IF analysis results revealed the co-localizations of pTDP-43 pathology with MAP-2 in M1 (Fig. 4a3) and parvicellular reticular nucleus (Fig. 4b3), ubiquitin in cervical spinal cord (Fig. 4c3), GFAP in LGP (Fig. 4d3), Iba-1 in medullary reticular nucleus and dorsal part (MdD) (Fig. 4e3). Neuronal cytoplasm contained the most abundant pTDP-43-positive aggregates (Fig. 4a3, b3), while few co-localizations of pTDP-43 with GFAP (Fig. 4d3) or Iba-1 (Fig. 4e3) were observed. Strong anti-ubiquitin signals were observed in neuron cytoplasm co-located with pTDP-43 (Fig. 4c3) in the Cs. Myelinolysis in the bilateral dorsal corticospinal tract was revealed by co-staining of MBP and NF (Fig. 4f1–g3). Obviously, the myelinolysis in the contralateral site was more pronounced than that in the ipsilateral site of TDP-43 PFFs injection. We semi-quantitatively detected the level of insoluble pTDP-43 over time in the cortex, RN, py, and cervical spinal cord of TDP-43 PFFs M1-C mice by WB. More insoluble pTDP-43 were detected in the extracts of TDP-43 PFFs M1-C mice compared with age-matched PBS M1-C mice at 4 mpi (Fig. 4h–o). In addition, we semi-quantitatively detected the insoluble C-terminal fragments of TDP-43 (CTFs) with time after injection in the cortex and Cs of TDP-43 PFFs M1-C and PBS M1-C mice. The WB analysis revealed increased levels of CTFs in both the cortex and Cs of TDP-43 PFFs M1-C mice at 4 and 8 mpi (Additional file 1: Fig. S7).

To detect whether TDP-43 PFFs M1-C mice developed age-dependent neuron loss, we performed stereology counts of Nissl staining in M1, ION, py and Cs in TDP-43 PFFs M1-C and PBS M1-C mice at 3 and 6 mpi, respectively. Furthermore, immunohistochemical staining for MAP-2 positive neurons in the M1, ION, py and calbindin (Cab) positive neurons in the Cs in TDP-43 PFFs M1-C mice at 3 and 6 mpi were also used to analyze the neuron loss. The findings indicated TDP-43 PFFs inoculation resulted in a increasingly significant decrease of neurons in TDP-43 PFFs M1-C mice as compared to PBS M1-C mice (Additional file 1: Fig. S8Aa-l and Fig. S8Ba-p). Moreover, we measured the brain wet weight of TDP-43 PFFs M1-C mice and PBS M1-C mice at 1, 3, 6, 8 mpi as reported previously [17]. The results showed that the TDP-43 PFFs M1-C mice presented obvious brain atrophy with age compared to the PBS M1-C mice (Additional file 1: Fig. S8C).

### Motor deficits in TDP-43 PFFs M1-C mice

**Fig. 5:**
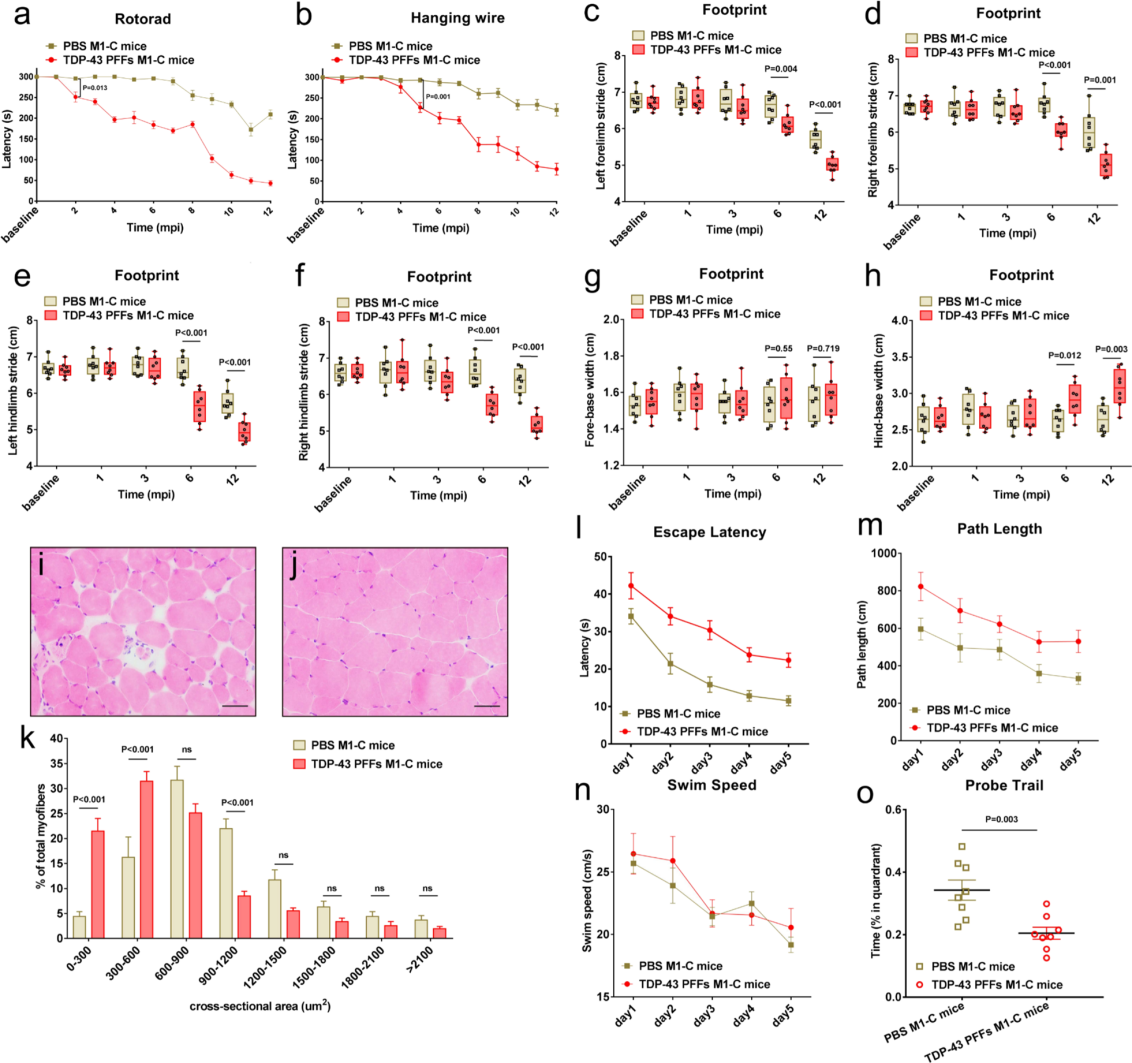
Behavioral analysis of TDP-43 PFFs M1-C and PBS M1-C mice. Rotarod test analysis (**a**) and hanging wire test (**b**) of mice at various time post injection. Footprint analysis of the left forelimb stride length (**c**), right forelimb stride length (**d**), left hindlimb stride length (**e**), right hindlimb stride length (**f**), fore-base width (**g**), hind-base width (**h**) at various time post injection. H&E staining for the right biceps brachialis muscles of TDP-43 PFFs M1-C mice (**i**) and PBS M1-C mice (**j**). Quantity of muscle fibers at different cross-sectional areas (**k**). Morris water maze analysis of escape latency (**l**), path length (**m**), swim speed (**n**), probe trial (**o**) at various time post injection. n = 9 mice/group. The error bar in all panels represents the Standard Error of Mean (SEM). Data are the mean ± SEM. Statistical analysis was performed using the Student’s t test.

In addition to the neuropathological evidence of TDP-43 PFFs-induced damage, we also investigated whether the TDP-43 PFFs-injected mice exhibited motor dysfunction. The lifespan among TDP-43 PFFs M1-C mice, PBS M1-C mice, Thy1-e (IRES-TARDBP) 1 mice and C57BL/6J mice showed no significant difference (Additional file 1: Fig. S3r). In addition, motor functions were evaluated by a series of behavioral tests, including rotarod test, hanging wire test, and footprint test once a month from 1 to 12 mpi in TDP-43 PFFs M1-C and PBS M1-C mice. The rotarod test revealed reduced movements in TDP-43 PFFs M1-C mice from 2 mpi compared with PBS M1-C mice, while the PBS M1-C mice showed no movement reduction compared with C57BL/6J mice even at 12 mpi (Fig. 5a). As shown in Fig. 5b, the hanging wire test revealed a progressive loss of muscle strength, motor coordination, and balance in TDP-43 PFFs M1-C mice compared with PBS M1-C mice at 5 mpi. For the footprint test, TDP-43 PFFs M1-C mice exhibited shorter stride length, wider base width, and lower speed than age-matched PBS M1-C mice as early as 6 mpi (Fig. 5c–h), which demonstrated gait disturbances. Moreover, we found that the hind-climb muscles of TDP-43 PFFs M1-C mice were too weak to lift the pelvis off ground and lead dragging of hind-climb during the later period of footprint test at 6 mpi. What’s more, we performed biopsy on both sides of biceps brachialis muscles in TDP-43 PFFs M1-C mice at 6 mpi. Hematoxylin and eosin (H&E) staining showed abnormal changes including grouped atrophy, round muscle fibers, and muscle fibers with central nuclei in the right biceps brachialis muscle (Fig. 5i), while no obvious morphological changes were found in the contralateral biceps brachialis muscle. The age-matched PBS M1-C mice were used as a negative control, as shown in Fig. 5j. Analysis of size distribution of the fiber cross-sectional areas in the right biceps brachialis muscles showed a decreased proportion of myofibers with relatively larger diameter (900-1200 µm^2^), and an increased proportion of myofibers with relatively smaller diameter (0-600 µm^2^) in TDP-43 PFFs M1-C mice compared with PBS M1-C mice (Fig. 5k).

### Learning and memory deficits in TDP-43 PFFs M1-C mice

In order to assess learning and memory, mice were tested in the Morris water maze. The escape latency in TDP-43 M1-C group was significantly longer than that in PBS M1-C group (Fig. 5l). Similar results were obtained when analyzing the path length to reach the platform (Fig. 5m). In addition, the swimming speed did not differ significantly among all groups (Fig. 5n). In the probe trial, spatial reference memory was strongly impaired in TDP-43 PFFs M1-C mice, and TDP-43 PFFs M1-C mice did not show a preference for the trained target quadrant in comparison with the PBS M1-C mice (Fig. 5o).

### ALS-like neurophysiological phenotypes in TDP-43 PFFs M1-C mice

**Fig. 6:**
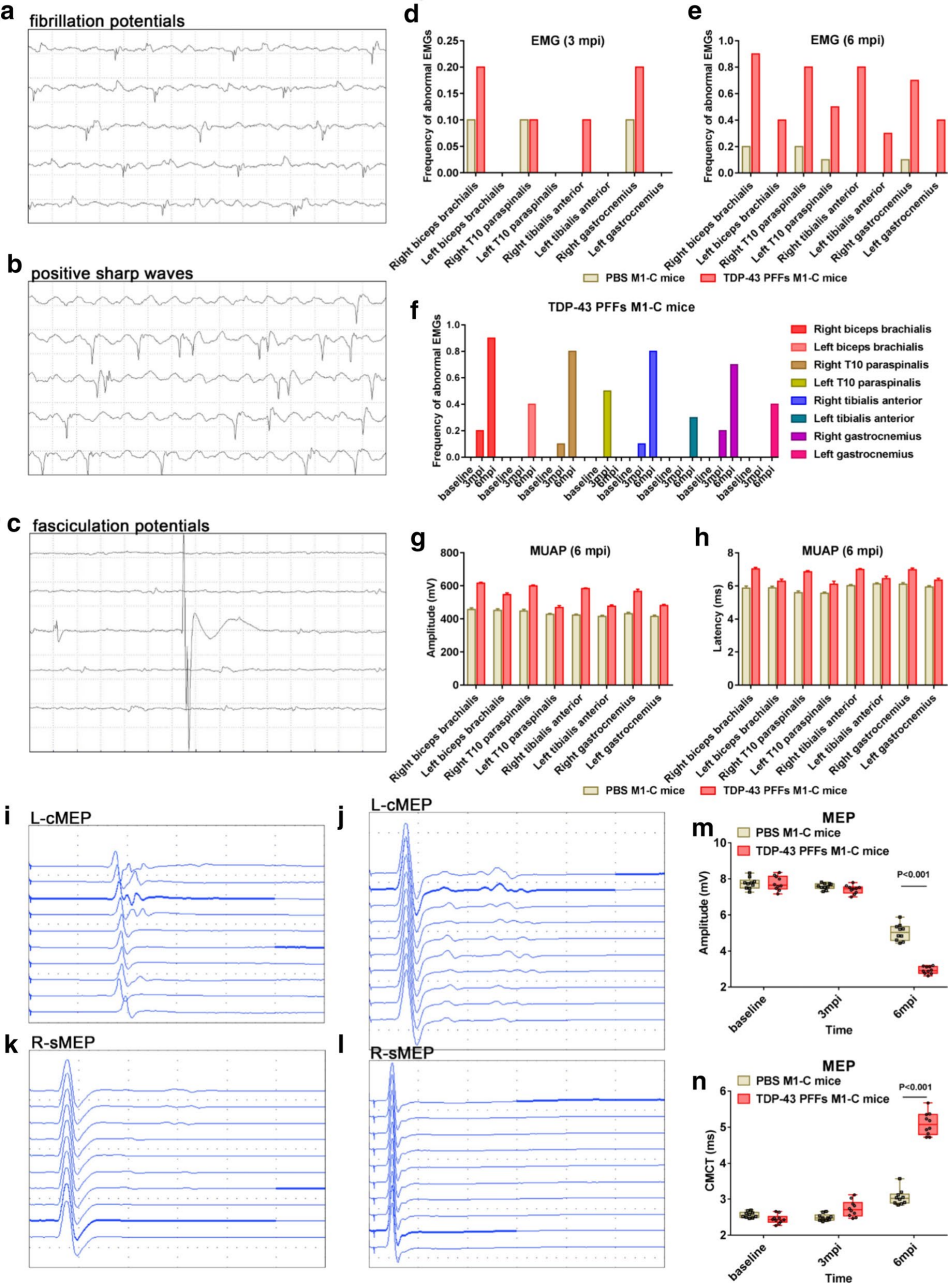
Neurophysiology of TDP-43 M1-C and PBS M1-C mice. **a–d** Spontaneous potentials including fibrillation potentials (**a**), positive sharp waves (**b**) and fasciculation potentials (**c**) in the right side of biceps brachialis, tibialis anterior, and gastrocnemius muscles were detected in TDP-43 PFFs M1-C mice at 3 mpi. The frequencies of spontaneous potentials in bilateral biceps brachialis, T10 paraspinals, tibialis anterior, and gastrocnemius muscles muscles were much higher in TDP-43 PFFs M1-C mice compared to PBS M1-C mice at 6 mpi (**e**). The frequency of abnormal spontaneous activity was developed in a time-dependent manner (**f**). The amplitude (**g**) and latency (**h**) of MUAPs were significantly increased in TDP-43 PFFs M1-C mice compared to PBS M1-C mice at 6 mpi, n = 10 mice/group, *P *< 0.05. Left-cMEP (L-cMEP) (**i**, **j**) and Right-sMEP (R-sMEP) (**k**, **l**) were detected in TDP-43 PFFs M1-C mice (**i**, **k**) and PBS M1-C mice (**j**, **l**). The amplitude of L-cMEP was decreased in TDP-43 PFFs M1-C mice compared to PBS M1-C mice at 6 mpi, n = 10 mice/group, *P *< 0.001 (**m**). The CMCT was increased in TDP-43 PFFs M1-C mice compared to PBS M1-C mice at 6 mpi, n = 10 mice/group, *P *< 0.001 (**n**). The error bar in all panels represents the Standard Error of Mean (SEM). Data are the mean ± SEM. Statistical analysis was performed using the Student’s t test

We next examined the electromyography (EMG) and motor evoked potentials (MEPs) of TDP-43 PFFs M1-C and PBS M1-C mice at 1, 3, 6, and 8 mpi. Spontaneous activity and motor unit action potentials (MUAPs) were used to quantify LMN dysfunction. MEPs were used to infer the mass activity of motor cortical neurons. Needle EMG was recorded from the bilateral biceps brachialis, tenth thoracic (T10) paraspinal, tibialis anterior, and gastrocnemius muscles. As early as 3 mpi, abnormal spontaneous activity including fibrillation potentials, fasciculation potentials, and positive sharp waves were detected in the right side of biceps brachialis, tibialis anterior, and gastrocnemius muscles of TDP-43 PFFs M1-C mice (Fig. 6a–d). Then, both sides of biceps brachialis, T10 paraspinals, tibialis anterior, and gastrocnemius muscles of TDP-43 PFFs M1-C mice were affected by abnormal spontaneous activity at 6 mpi (Fig. 6e). Nevertheless, in PBS M1-C mice, slight abnormal spontaneous activity emerged in bilateral biceps brachialis, T10 paraspinal, tibialis anterior, and gastrocnemius muscles at 8 mpi (Additional file 1: Fig. S9d). The frequency of abnormal spontaneous activity in TDP-43 PFFs M1-C mice was developed in a time-dependent manner, as shown in Fig. 6f. In addition, the motor unit measurement showed a significant increase in the mean amplitude and latency of MUAPs in bilateral biceps brachialis, T10 paraspinal, tibialis anterior, and gastrocnemius muscles at 6 mpi in TDP-43 PFFs M1-C mice, compared with age-matched PBS M1-C mice (Fig. 6g, h). Then, cortical MEP (cMEP) and spinal MEP (sMEP) were assessed to quantify upper motor neuron (UMN) impairment (Fig. 6i–l). At 6 mpi, L-cMEP amplitude decreased in TDP-43 PFFs M1-C mice compared with age-matched PBS M1-C mice (2.39 ± 0.34 mV versus 5.21 ± 0.24 mV, *P *< 0.001) at about 6 mpi (Fig. 6m). Moreover, central motor conduction time (CMCT) was significantly prolonged in the injection side in TDP-43 PFFs M1-C mice compared with age-matched PBS M1-C mice (4.49 ± 0.29 ms versus 3.05 ± 0.20 ms, *P *< 0.001), and the CMCT increased in a time-dependent manner (Fig. 6n). The statistical data of EMGs and MEP at all time points were shown in Additional file 1: Fig. S9. The above neurophysiological findings were served as preliminary evidence that TDP-43 PFFs injection caused ALS-like neurophysiological phenotypes.

### Dysphagia symptom in TDP-43 PFFs M1-L mice

To further investigate whether the TDP-43 PFFs M1-L mice would suffer dysphagia after TDP-43 PFFs injection, we evaluated the swallowing function of TDP-43 PFFs M1-L and PBS M1-L mice. As early as 5 mpi, TDP-43 PFFs M1-L mice showed significant weight loss (Additional file 1: Fig. S5d). At 6 mpi, the consumption of food and water showed a significant downward trend in TDP-43 PFFs M1-L mice compared with that in age-matched PBS M1-L mice (Additional file 1: Fig. S5e, f). Furthermore, statistical data showed substantial declines in both lick and mastication rates compared with age-matched PBS M1-L mice (Additional file 1: Fig. S5g, h).

## Methods

### Animals

The Thy1-e (IRES-TARDBP) 1 mouse line was constructed on a C57BL/6J background at Shanghai Bio-model Organisms Center, Inc. An IRES-TARDBP gene was knocked into the stop exon of the Thy1 gene via CRISPR/Cas9 strategy (Additional file 1: Fig. S10). The expression of human *TARDBP* gene was under the control of endogenous Thy1 promoter. To avoid off-target effects, knock-in mice were then backcrossed onto a C57BL/6J background for three generations. The genotype of mice was confirmed by PCR using the primers in Additional file 1: Table S1, heterozygote mice, male and female, were used in experiments. All mice were housed under standard conditions of constant temperature and controlled lighting (21 °C, 12/12 h light/dark cycle) and had food and water at libitum. All animal experiments have been approved by the Animal Care and Ethics Committee of Zhengzhou University.

### TDP-43 PFFs preparation and in vitro TDP-43 seeding assay

TDP-43 PFFs preparation from purified TDP-43 monomer was performed as reported previously [14, 22]. Briefly, human derived TDP-43 protein (ab156345, 1 mg/ml, purity: > 90%) purchased from Abcam Company was resuspended in double-distilled water at a concentration of 0.5 mg/ml, and then the samples were incubated at 25 °C with agitation at 1400 rpm for 2 h. Sequentially, the TDP-43 PFFs were sonicated by an ultrasonic cell disruptor at 10% of its peak amplitude (Scientz-IID, Ningbo, China) for 50 s and then frozen and stored in a − 80 °C freezer. To observe TDP-43 fibrils, ThT staining was performed as followed: 25 µM ThT solution was mixed with TDP-43 PFFs solution (0.5 mg/ml) by pipetting and incubated for 5 min at room temperature. Fluorescence (440 nm excitation, 480–1000 nm emission) was measured in a plate reader (ARVO MX, PerkinElmer Life Sciences). After ThT staining, we used a TEM to observe the nature of TDP-43 PFFs. 5 µl of the sample was applied on a hydrophilic, 200 mesh, carbon-coated copper grids (Yasheng Electronics Technology Co, Ltd., Zhengzhou, China) and then stained with 2% uranyl acetate. After washing with distilled water, the grids were allowed to air-dry. Images were obtained by transmission electron microscopy (JEOL USA, Inc., Peabody, MA, USA). Then, we mixed prepared TDP-43 PFFs with TDP-43 monomer solution, and treated the mixture with ThT fluorescence assay. The fluorescence was detected at intervals of 30 min for 7 days with 440 and 480 nm of excitation and emission wavelength. During the reactions, no plate shaking was performed. Additionally, the fibril nature of the TDP-43 PFFs was further confirmed by analysis of PK (2.0 µg/ml, Solarbio, China) resistance using a dot-blot test.

### Stereotactic injections

The mice were deeply anesthetized with isoflurane via intraperitoneal injection and fixed on a standard stereotaxic frame (David Kopf Instruments, Tujunga, CA, USA). Latch the front teeth into the anterior clamp and then tighten the clamp to ensure their heads were fixed firmly to the stereotaxic apparatus. After skin preparation, both the sagittal and coronal sutures of the skull were exposed and then we made a burr-hole just above the injection site with a metallic needle according to the scheduled coordinates. The two stereotaxic coordinates for M1 were as followed: M1-C: anteroposterior, 0.7 mm; mediolateral, 1.5 mm; dorsoventral, 1.4 mm; M1-L: anteroposterior, 2.4 mm; mediolateral, 1.5 mm; dorsoventral, 1.4 mm. The injection (5 μl per side) was performed at a rate of 0.2 µl/min using a 10-μl Hamilton syringe with a 34-gauge needle. In the present study, the Thy1-e (IRES-TARDBP) 1 mice injected with TDP-43 PFFs into the left M1-C and the left M1-L were named as TDP-43 PFFs M1-C and TDP-43 PFFs M1-L mice, respectively. Similarly, the Thy1-e (IRES-TARDBP) 1 mice injected with PBS into the left M1-C and the left M1-L were named as PBS M1-C and PBS M1-L mice, respectively.

### Immunohistochemistry and Immunofluorescent

For immunohistochemistry, brain and spinal cord from mice perfused transcardially by a 4% paraformaldehyde solution were fixed overnight in a mixture of 30% sucrose solution and 4% paraformaldehyde. Tissues embedded in paraffin blocks were cut into 4 μm-thick sections using the Rotary Microtome (Leica RM2235, Leica, Nussloch, Germany) and then the sections were deparaffinized in xylene and rehydrated. To eliminate endogenous peroxidase activity, tissues were incubated with 3% hydrogen peroxide solution for 10 min. The antigen-retrieval step was performed by putting tissues in citrate buffer (pH 6.0) for 10 min. Blocked with normal goat serum, primary antibodies were applied to each section overnight at 4 °C and then secondary antibodies were applied. We used Streptavidin-Peroxidase kit (Bioss, China) to detect the antigen–antibody complexes and visualized them with 0.05% 3,3′-diaminobenzidine (DAB, Neobioscience). For immunofluorescence labeling, sections were first blocked with normal goat serum and then incubated with primary antibodies overnight at 4 °C. After washed 3 × 5 min with PBS, sections were incubated with secondary antibodies at room temperature for 3 h. Nuclear staining was performed with Hoechst 33258 (1:1000, Solarbio). Finally, tissues were observed with an immunofluorescent microscopy. Serial coronal sections of the Cerebral Cortex, Hippocampi, midbrain, Medulla, cervical spinal cord and lumbar spinal cord were analyzed, especially the four representative sections: Bregma 0.74 mm, Bregma − 1.70 mm, Bregma − 3.80 mm, and Bregma − 7.64 mm. Commercially available antibodies used in this study are shown in Additional file 1: Table S2. The immunohistochemical images were processed using ImageJ software (US National Institutes of Health) and the percentage of areas occupied by pTDP-43 staining was quantified.

### Western blots

To perform western blots, tissues from brain, spinal cord of 4-mpi TDP-43 PFFs M1-C mice were cut up and separated into detergent-soluble and -insoluble fractions by RIPA buffer (150 mm NaCl; 1 mm EDTA, pH 8.0; 50 mm Tris, pH 8.0; 0.5% DOC and 1% Triton X-100; 1 mm EGTA, pH 8.0; 0.1% SDS), and complete protease inhibitor mixture (Thermo Fisher Scientific, U.S.A). After 30 min of ultracentrifugation at 100,000*g* at 4 °C, the resulting supernatants representing the soluble fractions were collected, and then, the pellets were further rinsed in RIPA buffer (Beyotime, China) by 30 min of ultracentrifugation at 100,000*g* at 4 °C two times in a row. The final pellets representing the insoluble fractions were solubilized in 8 M urea buffer. 10 μg of each fraction was loaded and electrophoresed on 15% SDS–polyacrylamide gel and then transferred to a polyvinylidene fluoride membrane. To detect target proteins, blocked with 5% nonfat dry milk in TBS at RT for 1 h, the membranes were then treated with primary antibodies for 48 h. Membranes were rinsed and washed in TBST (TBS with 0.1% Tween-20) for 3 × 10 min. The second incubation was performed with an HRP-conjugated secondary antibody at room temperature for 2 h and visualized by enhanced chemiluminescence (ECL, Thermo Fisher Scientific, U.S.A). Proteins on the blots were normalized to GAPDH (mouse, Millipore, 1:2000). Quantification of the immunoblotted bands was assessed using the FluorChem 8900 software (Alpha Innotech, San Leandro, CA, USA).

### Heat maps

The semiquantitative heat maps for TDP-43 PFFs M1-C mice were generated using stained slides with p409-410 antibody. Briefly, pTDP-43 pathology was graded from negative (0, gray) to more abundant (3, red) and color-coded into six representative coronal sections from rostral to caudal brain levels and spinal cord (Bregma: 0.74 mm, − 1.70 mm, − 3.80 mm, − 7.64 mm, C7 (the seventh cervical spinal cord segment), L3 (the third lumbar spinal cord segment)). Brain regions were marked according to the anatomical patterns in The Mouse Brain Atlas in stereotaxic coordinates [28]. After grading individual brain regions in each mouse, averaged values for each time point (1 mpi (n = 3), 2 mpi (n = 3), 4 mpi (n = 3), and 8 mpi (n = 3)) were imported into CNS heat maps using the in-house software to create the distribution maps of pTDP-43 pathology based on a scale color system for Alzheimer’s disease (AD) [18].

### Nissl staining

For Nissl staining, brains from mice perfused transcardially by 4% paraformaldehyde solution were fixed overnight in a mixture of 30% sucrose solution and 4% paraformaldehyde. Tissues embedded in paraffin blocks were cut into 4 μm-thick sections using the Rotary Microtome (Leica RM2235, Leica, Nussloch, Germany) and then the sections were deparaffinized in xylene and rehydrated. Then, the slices were placed in Cresyl violet Stain and dipped the dye vat in a 56 °C incubator for 1 h. After rinsing with deionized water, the slices were placed in Nissl Differentiation for 20 s to 2 min until the background was close to colourlessness under the microscope.

### H&E staining

To perform H&E staining, we collected bilateral biopsied biceps brachialis muscles from TDP-43 PFFs M1-C mice and PBS M1-C mice at 4 mpi. Then the tissues were rinsed in isopentane immediately and cooled in liquid nitrogen. Tissues were cut into 10 μm-thick sections and then sections were stained by hematoxylin and eosin. All the steps we performed above were in accordance with the standard procedure [32].

## Behavioral studies

### Motor function assessment

#### Rotarod test

For the assessment of motor coordination and balance, we used a rotarod apparatus (Rotarod YLS-4C; YiYan Science and Technology Development Co., Ltd. Shandong, China). Briefly, each mouse was placed on the rotarod that was rotating at 30 rpm. The maximal latency to fall off was collected for statistical analysis. Before the testing day, each mouse first received three trials per day for five consecutive days. On the test day, each mouse was given three trials with a 10 min interval. For each trial, every mouse was tested three times and the maximal latency (maximum of 5 min) was recorded. The average of the three trials was calculated and recorded for each mouse.

#### Hanging wire test

The wire-hanging test evaluates neuromuscular grip strength and motor function of each mouse. All the PBS or TDP-43 PFFs M1-C mice were individually placed on a standard wire cage lid. The test was performed by placing the mouse on the top of a wire cage lid. The time to drop off from the wire (latency to fall) of each mouse was noted. Three trials were performed for each mouse. During each trial, mice were tested three times and the best performance of each mouse was recorded. The average of the three trials was calculated and used for data analysis.

#### Footprint test

Gait was assessed using the footprint test. After coating feet with a nontoxic paint (fore-paws in red and hind-paws in green), mice were placed on a restricted cardboard tunnel (50 cm long, 5 cm wide, 10 cm high) and allowed to move freely across the walkway. A fresh sheet of white paper (42 cm long, 4.5 cm wide) was placed on the floor of the walkway, and the footprints of each mouse were collected. Three uninterrupted footsteps from the middle portion of each run were measured for the following parameters (cm): (1) stride length (front and hind legs). (2) The front- and hind-base width. The mean value of each set of outcomes was calculated for statistical analysis.

### Learning and memory function assessment

#### Morris water maze

To assess spatial and related abilities of learning and memory, the Morris water maze test was performed as previously described [43]. Morris’ water test was performed in a 2 m-diameter circular pool divided into four quadrants and filled with water made opaque by non-toxic titanium dioxide. The temperature in the pool was maintained at 22 °C. A 10 cm-diameter escape platform was placed in the target quadrant 0.5 cm beneath the water surface. Mice were trained 4 times a day continuously for 5 days. In each trial, mice were released pseudo-randomly from assigned start locations (north, south, east or west) and maximum swim time was set to 60 s. Mice failing to find the platform were manually guided to the platform. Mice succeeding in finding the platform were left on the platform for 15 s before being dried and returned to their cages. 24 h after the last training day, single probe trials to test reference memory were conducted by removing the platform. The platform was removed then the mice were released at the opposite position and were allowed to swim for 60 s. Animals were video tracked using Ethovision software (Noldus, Wageningen, the Netherlands) and behavioral parameters (escape latency, path length, swim speed, percentage of time spent in each quadrant) were automatically measured.

### Swallowing function assessment

The assessment was performed as previously reported [31]. Direct measures of dysphagia were carried out by lick and mastication rates. Before the test, each mouse was individually housed in a transparent cage and a restricted water and food schedule was performed for a week to motivate mice to drink and eat in a regular period. 24 h before test day, food and water were completely removed away so that mice can’t get any food and water. On the test day, each mouse was provided with tap water in a water bottle and food on the floor of the cage. Eating and drinking behaviors of mice were video recorded for 10 min and then each mouse was taken back to its previous cage with free access to food and water. Lick rates (number of rhythmic licks per second) and mastication rates (number of rhythmic chewing cycles per second) were recorded by a video camera. Indirect assessments of dysphagia were performed by calculation of body weight and food and water consumption. Body weight of each mouse was recorded every week after surgery. In each week, we gave 100 g food and 300 mL tap water to each mouse on Monday and at the same time next week we weighed remanent food and water and the consumption of food and water was recorded.

### Electrophysiological examination

#### EMGs

Mice were anesthetized with isoflurane and then fixed on a platform in the prone position. To place the EMG electrodes, after skin preparation at the positions corresponding to the muscles we detected (bilateral biceps brachialis muscles, bilateral T10 paraspinal muscles, bilateral tibialis anterior muscles, and bilateral gastrocnemius muscles). All the electrodes were placed in the thick portion of each muscle. To make sure that the mouse body was electrically grounded, a needle electrode was implanted in the abdominal wall. MUAPs and spontaneous activities including fasciculation potentials and fibrillation potentials were collected to assess the function of UMN and LMN. In detail, 20 MUAPs were recorded at different parts and spontaneous activities were recorded at least 120 s. Apparatus we used in this test were as followed: a standard EMG device (MEB-2306C, Nihon Kohden Corporation, Tokyo, Japan) and some concentric needles (25.0 mm × 0.3 mm, Technomed Europe, Beek, Netherlands).

### MEPs induced by electrical stimulation

MEPs including cMEP and sMEP were obtained by electrical stimulation with the same apparatus. To implant electrodes, incisions approximate 1.5 cm on the scalp and the neck of mice were made. For the epicranial electrode implantation, the anode was positioned over the motor cortex, approximate 2 mm right to bregma and the cathode was placed at the midline of the interaural line. For sMEP, electrodes were placed into the C7. The ground electrode was inserted into the tail. With the active electrode inserted in the muscle of the forelimb footpad and the reference needle electrode placed under the skin of the second digit, cMEP and sMEP were recorded. CMCT, a parameter of the propagation time along corticospinal tracts, was calculated as the difference between the cMEP latency and the sMEP latency.

### Statistical analysis

All data were quantified and expressed as mean ± SEM. Statistical analysis for all data was performed using SPSS 24.0 (IBM, Armonk, New York, USA). Mice were divided into the TDP-43 PFFs M1-C mice and PBS M1-C mice, TDP-43 PFFs M1-L mice and PBS M1-L mice, untreated Thy1-e (IRES-TARDBP) 1 mice and C57BL/6J mice. Unpaired Student’s t test was performed to compare the behavioral evaluation, IHC and WB results between PFFs-injected mice and PBS-injected mice. Data from IHC and negative-stained transmission electron micrographs were analyzed by ImageJ software (US National Institutes of Health). P ≤ 0.05 indicates that the difference between the groups is statistically significant. All statistical graphs were created by Prism software 8.0 (GraphPad Software, La Jolla, CA).

## Discussion

A considerable number of in vitro studies have confirmed that pathological TDP-43 could transmit from cell to cell along axons in a self-templating manner [13, 20, 34, 39]. Furukawa et al. [14] reported that sarkosyl-insoluble TDP-43 fibrils were able to elicit TDP-43 aggregation in HEK293T cells, and this seeding reaction can reproduce ubiquitinated TDP-43 aggregates in cells. Moreover, Nonaka et al. [34] found that sarkosyl-insoluble TDP-43 from ALS or FTLD brains could induce TDP-43 aggregation in SH-SY5Y cells, and these cells subsequently formed ubiquitinated, phosphorylated, insoluble cytoplasmic TDP-43 inclusions in a self-templating manner. In addition, TDP-43 oligomers may spread intercellularly across axon terminals of primary cortical mouse neurons [13]. Nevertheless, synthetic fibrils made from pure recombinant TDP-43 protein had not been demonstrated to induce TDP-43 pathology in vivo. Using solely pure recombinant TDP-43 protein, we synthesized TDP-43 fibrils with the ability to promote fibrillization of surrounding monomeric TDP-43, which was confirmed using ThT fluorescence assay, PK resistance analysis, and electron microscopy as previously described [22]. We found that not soluble but aggregated TDP-43 increased the fluorescence intensity of ThT. ThT has been confirmed to give strong fluorescence upon binding to amyloids [35, 38, 44]. It is worth noting that the TDP-43 fibrils prepared in this study showed stronger fluorescence intensity of ThT than the previous studies [14, 21]. We thus speculate that purified full-length TDP-43 protein may be more likely to form stable β-sheet structures, which is the main structure in amyloids binding to ThT. However, the increase in its fluorescence intensity seemed slightly smaller in TDP-43 aggregates than that in other protein aggregates, such as SOD1 [15]. These results were in line with the previous findings that pathological TDP-43 inclusions were not well stained by amyloid binding dyes [26], although their morphology was expected to be fibrillar. According to the TDP-43 PFFs seeding assay by ThT staining and WB, we confirmed the seeding property of synthetic TDP-43 PFFs in vitro. In addition, we have confirmed fibrillar morphologies of TDP-43 PFFs by electron microscopic observation.

Accumulating evidence has demonstrated that prion-like propagation of deformed proteins might underlie the pathophysiology in several noninfectious neurodegenerative diseases, including ALS, AD, and Parkinson’s disease (PD) [1, 10, 12, 23, 24, 27, 29, 30, 34]. Aggregates of TDP-43 that appear in both upper and lower motor neurons, and some glial cells are major pathologic feature of ALS [42]. Biochemical and histological analyses also demonstrated the accumulation of full-length TDP-43 and CTFs in hyperphosphorylated and fibrillar forms in the brain and spinal cord of patients with ALS and FTLD [19, 33]. According to the distribution of intraneuronal pTDP-43 aggregates in a large cross-sectional cohort of phenotypically well-defined ALS autopsy cases, the progression of ALS pathology can be divided into four stages [5, 7], which is consistent with the induction and dissemination of pTDP-43 pathology chiefly from cortical neuronal projections, via axonal transport, through synaptic contacts to the spinal cord [5]. Furthermore, an in vivo study showed that inoculation of brain extracts containing abundant pathological TDP-43 from FTLD-TDP patients can induce de novo TDP-43 pathology in TDP-43 transgenic mice expressing mislocalized cytoplasmic TDP-43 [36]. The pyramidal tract is the downward motion conduction tract including the corticospinal tract and the corticobulbar tract.

In this study, we used TDP-43 PFFs M1-C and TDP-43 PFFs M1-L mice models to study the spreading of pathological TDP-43 along the corticospinal tract and corticobulbar tract, respectively. Our results suggested that TDP-43 pathology might originate from M1, then spread through the pyramidal tract to the associated cortical projection neurons, which supported the Braak’s hypothesis. Previous studies failed to demonstrate whether transmission of TDP-43 pathology along pyramidal tract could induce ALS-like phenotypes. Our present study indicated that the TDP-43 transgenic mice injected with TDP-43 fibrils in M1 could develop pTDP-43 pathology along pyramidal tract and ALS-like phenotypes in a time dependent manner, which partly imitated the disease process of ALS. The primary feature of ALS is the degeneration of both UMN and LMN, leading to the gradual installation of motor deficits that develop within weeks or months [8, 42]. Loss of these neurons leads to a deterioration of neuromuscular function, causing weakness, atrophy and paralysis of skeletal muscles. Accordingly, we further assessed a wide range of behavioral phenotypes in this model, including the wire hanging test for general muscle strength, the rotarod assay for motor coordination, the footprint analysis for gait stability, and the water maze for the spatial learning and memory. Furthermore, the oral dysfunction was evaluated by the direct evidence (i.e., lick and mastication rate) and indirect evidence (i.e., body weight and food and water consumption). As a consequence, we found that TDP-43 PFFs M1-C mice gradually developed motor dysfunction, gait abnormalities, and cognitive impairment, while TDP-43 PFFs M1-L mice developed swallowing disorders in a time-dependent manner after TDP-43 fibrils injection. It is important to note that the diagnosis of ALS is based principally on clinical examination combining with electrophysiological investigation [11, 16]. Hence, we used EMG to determine locomotion mode, and MEPs to evaluate potential impairment of the pyramidal tract. As predicted, TDP-43 PFFs M1-C mice showed abnormal electrophysiological manifestations, revealing both upper and lower motor neuron lesions. These findings were consistent with the TDP-43 pathology distribution.

Taken together, our findings provide direct evidence that injection of fibrillar TDP-43 in the primary motor cortex could induce TDP-43 pathology in different brain regions and spinal cord via pyramidal tract in a stereotypic manner. Furthermore, this new model presents ALS-like pathological and behavioral phenotypes, which implicates in the possible pathogenesis of ALS.

## Supplementary Information

**Additional file 1.Supplementary Information. Fig. S1**: Distribution and expression level of hTDP-43 in different brain regions of Thy1-e (IRES-TARDBP) 1 mice and expression level of total TDP-43 in the cortex and spinal cord of different mice. **Fig. S2**: Lacking of pTDP-43 pathology in CNS segments of PBS M1-C mice. **Fig. S3**: Behavioral analysis of PBS M1-C mice, Thy1-e (IRES-TARDBP) 1 mice and C57BL/6J mice. **Fig. S4**: TDP-43 PFFs (sonicated) injection into M1-C or M1-L of mice. **Fig. S5**: TDP-43 pathology distribution in medulla oblongata and swallowing function analysis of TDP-43 PFFs M1-L mice. **Fig. S6**: Lacking of pTDP-43 pathology in CNS segments of TDP-43 PFFs M1-C C57BL/6J mice.  **Fig. S7**: The expression level of C-terminal TDP-43 between TDP-43 PFFs M1-C mice and PBS M1-C mice.  **Fig. S8**: Immunohistochemistry and Nissl Staining in different brain regions of TDP-43 PFFs M1-C mice and PBS M1-C mice and the analysis of brain weight. **Fig. S9**: Neurophysiology of TDP-43 PFFs M1-C mice and PBS M1-C mice. **Fig. S10**: IRES-TARDBP gene knocked into the stop exon of the Thy1 gene via CRISPR/Cas9 strategy. **Supplementary Table 1**: Primers used to identify the genotype of Thy1-e(IRES-TARDBP) 1 mice. **Supplementary Table 2**: Antibodies used in the study.

## Data Availability

All relevant data supporting the findings of this study are either included within the article and its Supplementary Information files or are available upon request from the corresponding author.

## Abbreviations

ALS: Amyotrophic lateral sclerosis
CA1: Field CA1 of hippocampus
CA2: Field CA2 of hippocampus
CA3: Field CA3 of hippocampus
cg: Cingulum
cMEP: Cortical MEP
CMCT: Central motor conduction time
Cs: Cervical spinal cord anterior horn
CTFs: C-terminal fragments of TDP-43
EMG: Electromyography
FTLD-TDP: Frontotemporal lobar degeneration with TDP-43 pathology
GFAP: Glial fibrillary acidic protein
hTDP-43: Human TDP-43
H&E: Hematoxylin and eosin
Iba-1: Ionized calcium binding adapter molecule 1
IC: Internal capsule
IF: Immunofluorescence
IHC: Immunohistochemistry
ION: Inferior olive nucleus
LGP: Lateral globus pallidus
LMN: Lower motor neuron
Ls: Lumber spinal cord anterior horn
M1: Primary motor cortex
M2: Secondary motor cortex
M1-C: Left caudal part of M1 region
M1-L: Left lateral part of M1 region
MAP-2: Microtubule-associated protein-2
MBP: Myelin basic protein
MdRt: Medullary reticular formation
MEPs: Motor evoked potentials
Mol: Molecular layer of the dentate gyrus
mTDP-43: Mouse TDP-43
mpi: Month post-injection
MUAPs: Motor unit action potentials
PK: Proteinase K
pTDP-43: Phosphorylated TDP-43
pTDP-43-ir: pTDP-43-immunoreactive
py: Decussatio pyramidum
RN: Red nucleus
Rt: Reticular thalamic nucleus
S1: Primary somatosensory cortex
S2: Secondary somatosensory cortex
sMEP: Spinal MEP
SN: Substantia nigra
st: Stria terminalis
STh: Subthalamic nucleus
TDP-43: Transactive response DNA-binding protein 43 kDa
TDP-43 PFFs: TDP-43 preformed fibrils
TEM: Transmission electron microscope
ThT: Thioflavin T
Ub: Ubiquitin
UMN: Upper motor neuron
WB: Western blot
12N: Hypoglossal nucleus
