# Supplementary material for: Spreading of TDP-43 pathology via pyramidal tract induces ALS-like phenotypes in TDP-43 transgenic mice
Source: Acta Neuropathol Commun. 2021 Jan 18;9:15. doi: 10.1186/s40478-020-01112-3 (PMC7814549; doi:10.1186/s40478-020-01112-3)
Supplement: Supplementary file 1 — Additional file 1.Supplementary Information. Fig. S1: Distribution and expression level of hTDP-43 in different brain regions of Thy1-e (IRES-TARDBP) 1 mice and expression level of total TDP-43 in the cortex and spinal cord of different mice. Fig. S2: Lacking of pTDP-43 pathology in CNS segments of PBS M1-C mice. Fig. S3: Behavioral analysis of PBS M1-C mice, Thy1-e (IRES-TARDBP) 1 mice and C57BL/6J mice. Fig. S4: TDP-43 PFFs (sonicated) injection into M1-C or M1-L of mice. Fig. S5: TDP-43 pathology distribution in medulla oblongata and swallowing function analysis of TDP-43 PFFs M1-L mice. Fig. S6: Lacking of pTDP-43 pathology in CNS segments of TDP-43 PFFs M1-C C57BL/6J mice. Fig. S7: The expression level of C-terminal TDP-43 between TDP-43 PFFs M1-C mice and PBS M1-C mice. Fig. S8: Immunohistochemistry and Nissl Staining in different brain regions of TDP-43 PFFs M1-C mice and PBS M1-C mice and the analysis of brain weight. Fig. S9: Neurophysiology of TDP-43 PFFs M1-C mice and PBS M1-C mice. Fig. S10: IRES-TARDBP gene knocked into the stop exon of the Thy1 gene via CRISPR/Cas9 strategy. Supplementary Table 1: Primers used to identify the genotype of Thy1-e(IRES-TARDBP) 1 mice. Supplementary Table 2: Antibodies used in the study. [file 40478_2020_1112_MOESM1_ESM.docx]

**Supplementary Information**

**Supplementary Figures**


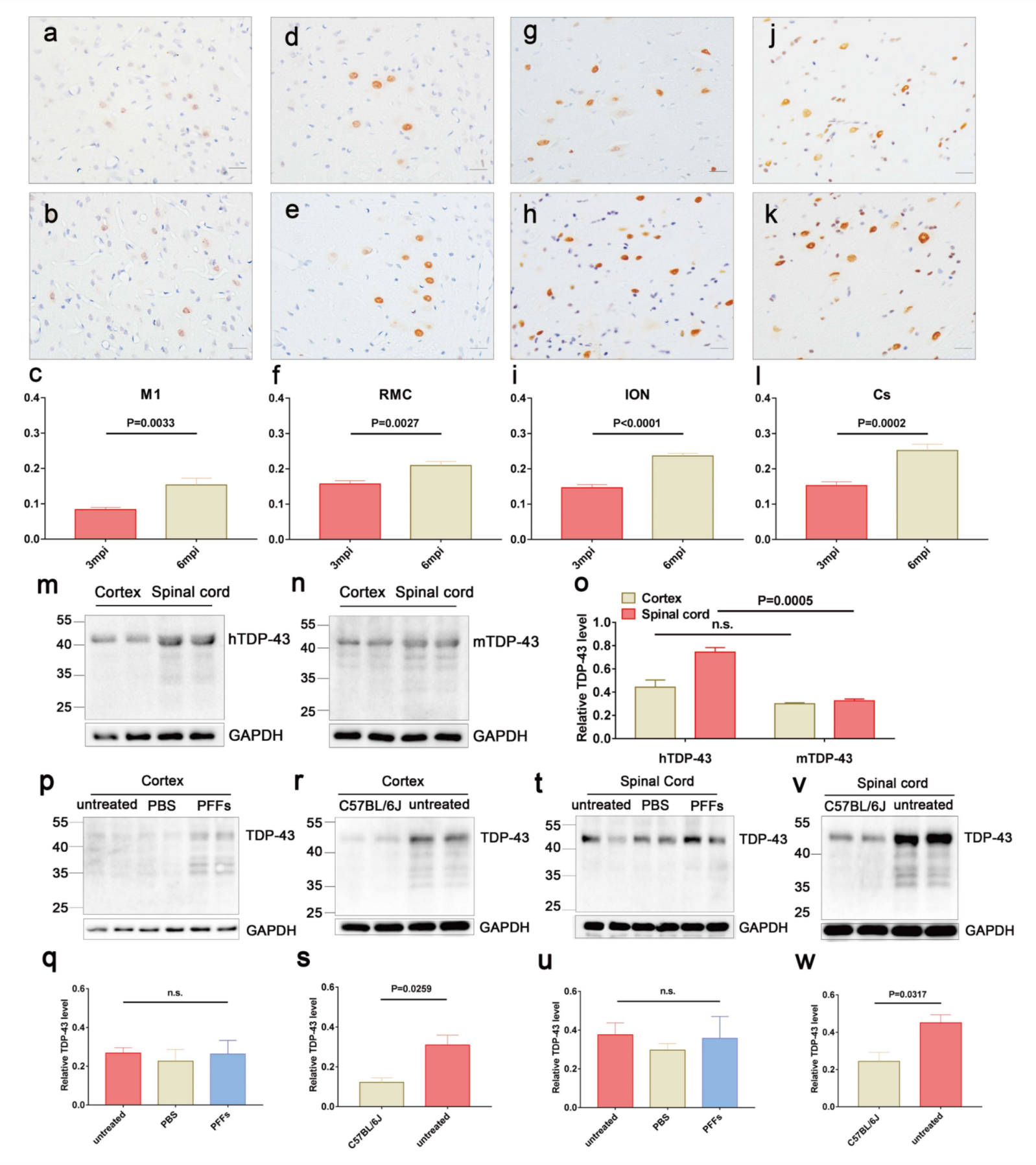


**Fig. S1. Distribution and expression level of hTDP-43 in different brain regions of Thy1-e (IRES-TARDBP) 1 mice and expression level of total TDP-43 in the cortex and spinal cord of different mice. (a-l)** Representative images of hTDP-43 immunoreactivity in M1 at 3 mpi **(a)**, 6 mpi **(b)** and stereology quantification **(c)**, RMC at 3 mpi **(d)**, 6 mpi **(e)** and stereology quantification **(f)**, ION at 3 mpi **(g)**, 6 mpi **(h)** and stereology quantification **(i)**, Cs at 3 mpi **(j)**, 6 mpi **(k)** and stereology quantification **(l)** of Thy1-e (IRES-TARDBP) 1 mice. n = 3 mice/age/group. **(m-o)** Representative WB images of hTDP-43 **(m)** and mTDP-43 **(n)** in the soluble fractions of cortex and spinal cord of Thy1-e (IRES-TARDBP) 1 mice at 4 mpi and quantification **(o)**. n = 3 mice/age/group. **(p-w)** Representative WB images of total TDP-43 in the soluble fractions of cortex **(p, r)** and spinal cord **(t, v)** of untreated Thy1-e (IRES-TARDBP) 1 mice, PBS M1-C mice, TDP-43 PFFs M1-C mice at 4 mpi and age-matched C57BL/6J mice and related quantification **(q, s, u, w)**. Blots were probed for GAPDH as a loading control (Bottom). n = 3 mice/age/group. Data are the means ± SEM. Statistical significance was analyzed using the Student’s t test and Mann-Whitney test. [Scale bars, a-k, 20 µm].

**
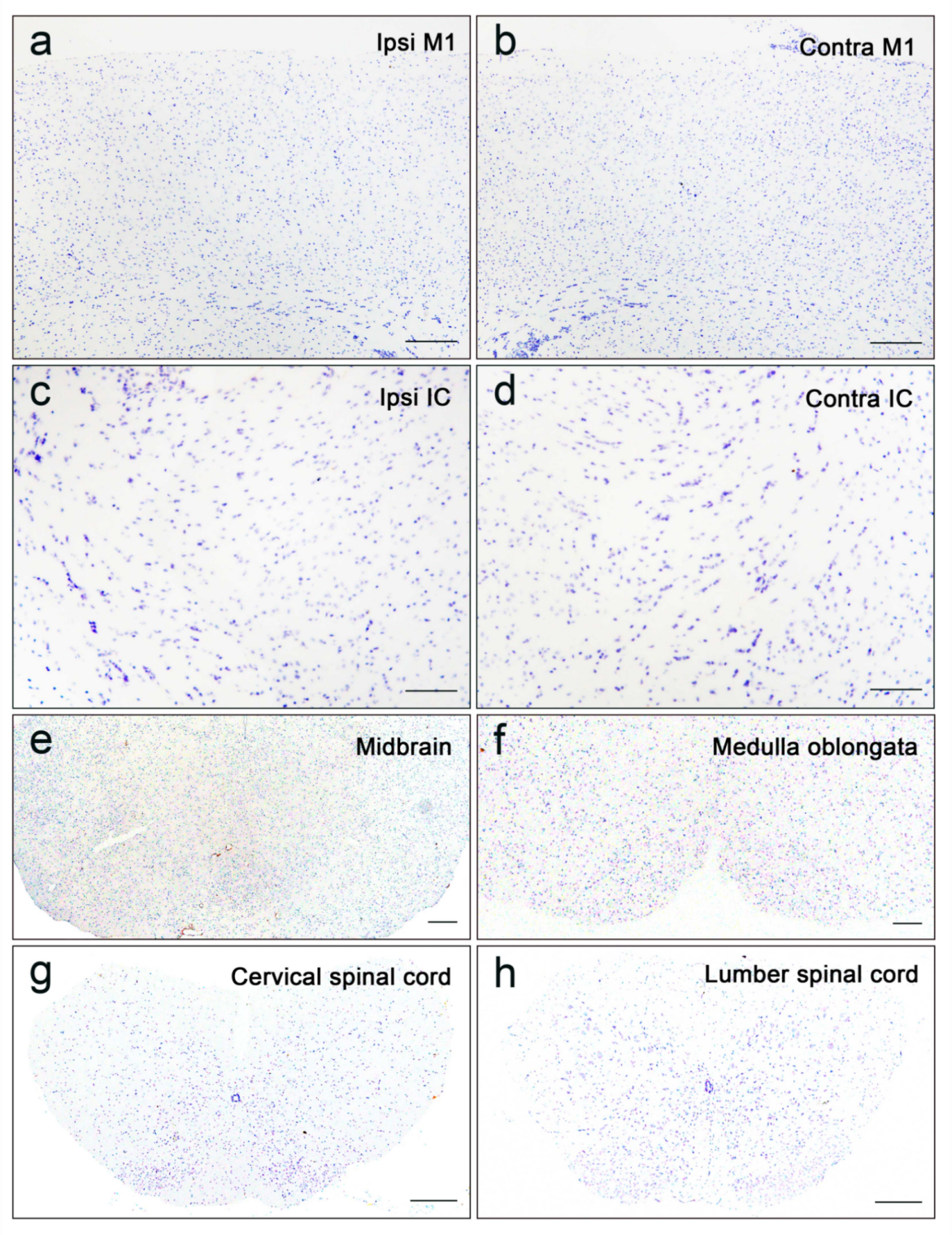
**

**Fig. S2.** **Lacking of pTDP-43 pathology in CNS segments of PBS M1-C mice.** Representative immunohistochemical results displaying the lacking of pTDP-43 pathology in the M1 **(a-b**), internal capsule (IC, **c-d**), midbrain (**e**), medulla oblongata (**f**), cervical spinal cord (**g**) and lumber spinal cord (**h**) from PBS M1-C mice at 8 mpi (n = 3 mice/group) in the ipsilateral (Ipsi) or contralateral (Contra) side of injection.


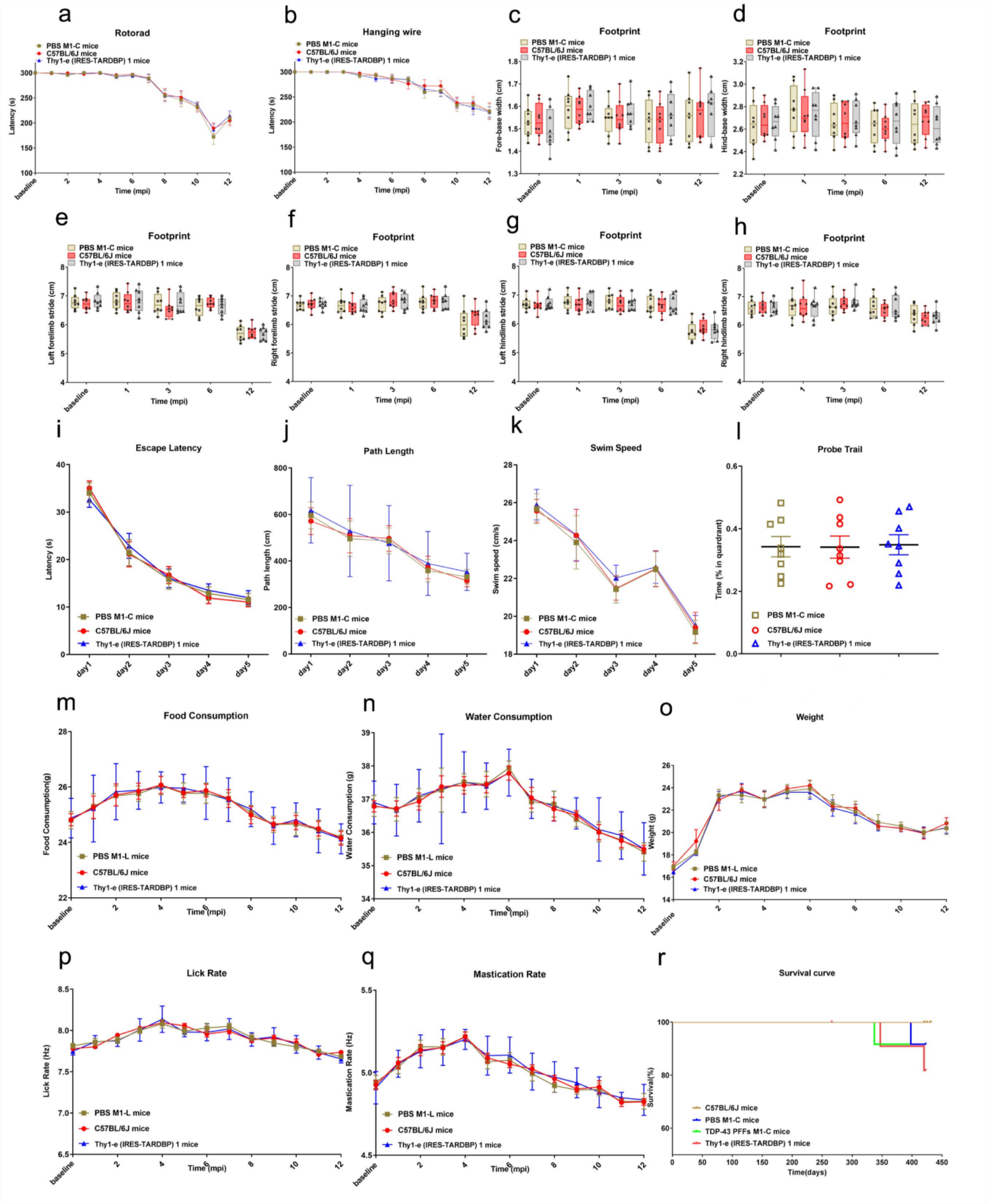


**Fig. S3. Behavioral analysis of PBS M1-C mice, Thy1-e (IRES-TARDBP) 1 mice and C57BL/6J mice.** Rotarod test analysis **(a)** and hanging wire test **(b)** of different mice at various time post injection. n = 6 mice/group. Footprint analysis of fore-base width **(c)**, hind-base width **(d)**, left forelimb stride length **(e)**, right forelimb stride length **(f)**, left hindlimb stride length **(g)**, right hindlimb stride length **(h)** of different mice at various time post injection. n = 8 mice/group. Morris water maze analysis of escape latency **(i)**, path length **(j)**, swim speed **(k)**, probe trial **(l)** of different mice at various time post injection. n = 8 mice/group. Indirect assessments of dysphagia of different mice performed by calculation of food consumption **(m)**, water consumption **(n)** and body weight **(o)**. Direct measures of dysphagia carried out by lick rates **(p)** and mastication rates **(q)**. n = 9 mice/group. The survival curve of C57BL/6J mice, PBS M1-C mice, TDP-43 PFFs M1-C mice and Thy1-e (IRES-TARDBP) 1 mice**(r)**. n = 12 mice/group. The error bar in all panels represents the Standard Error of Mean (SEM). Data are the means ± SEM. The data were statistically analyzed by the Student’s t test and Mann-Whitney test.

**
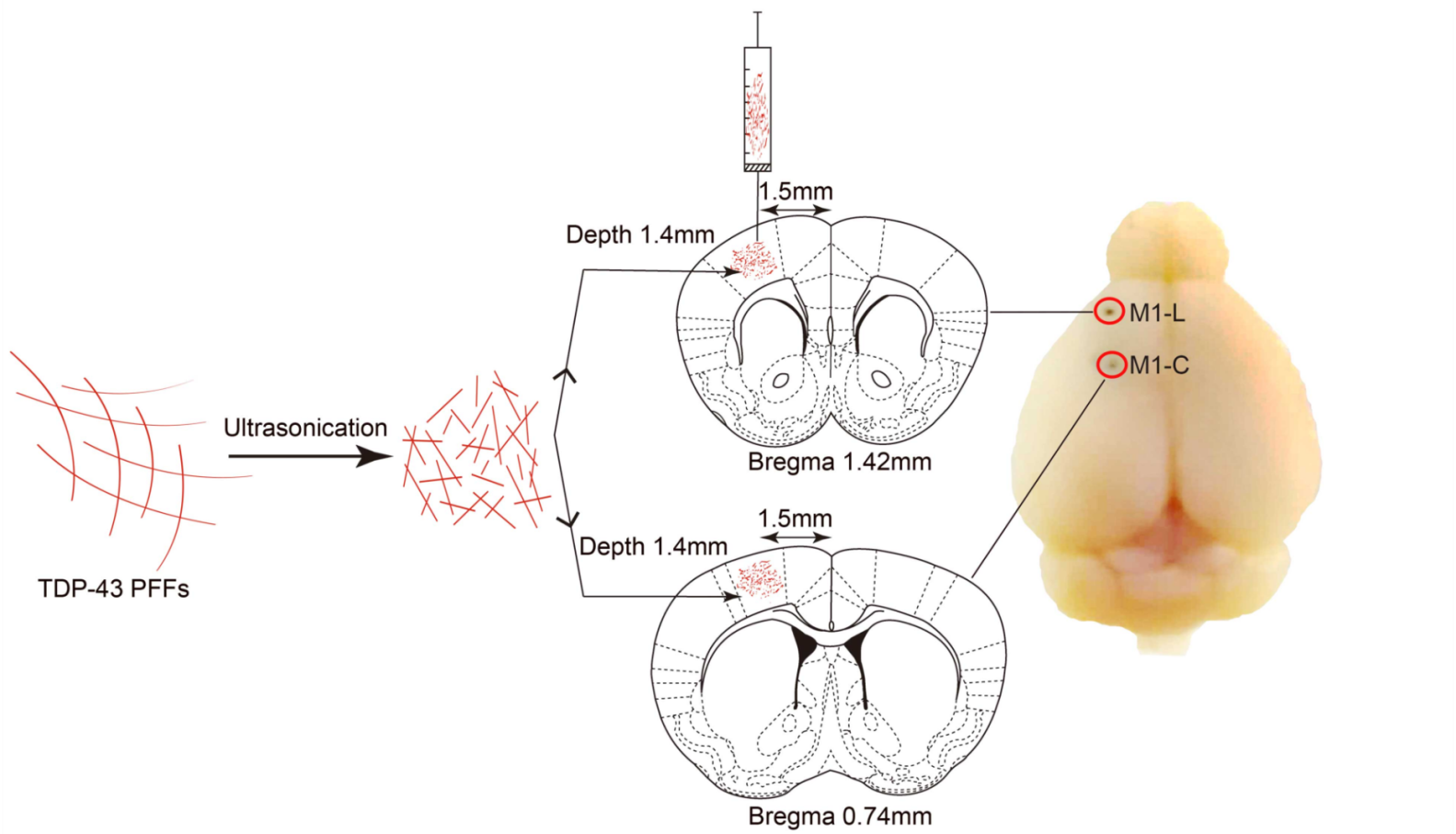
**

**Fig. S4. TDP-43 PFFs (sonicated) injection into M1-C or M1-L of mice.**

**
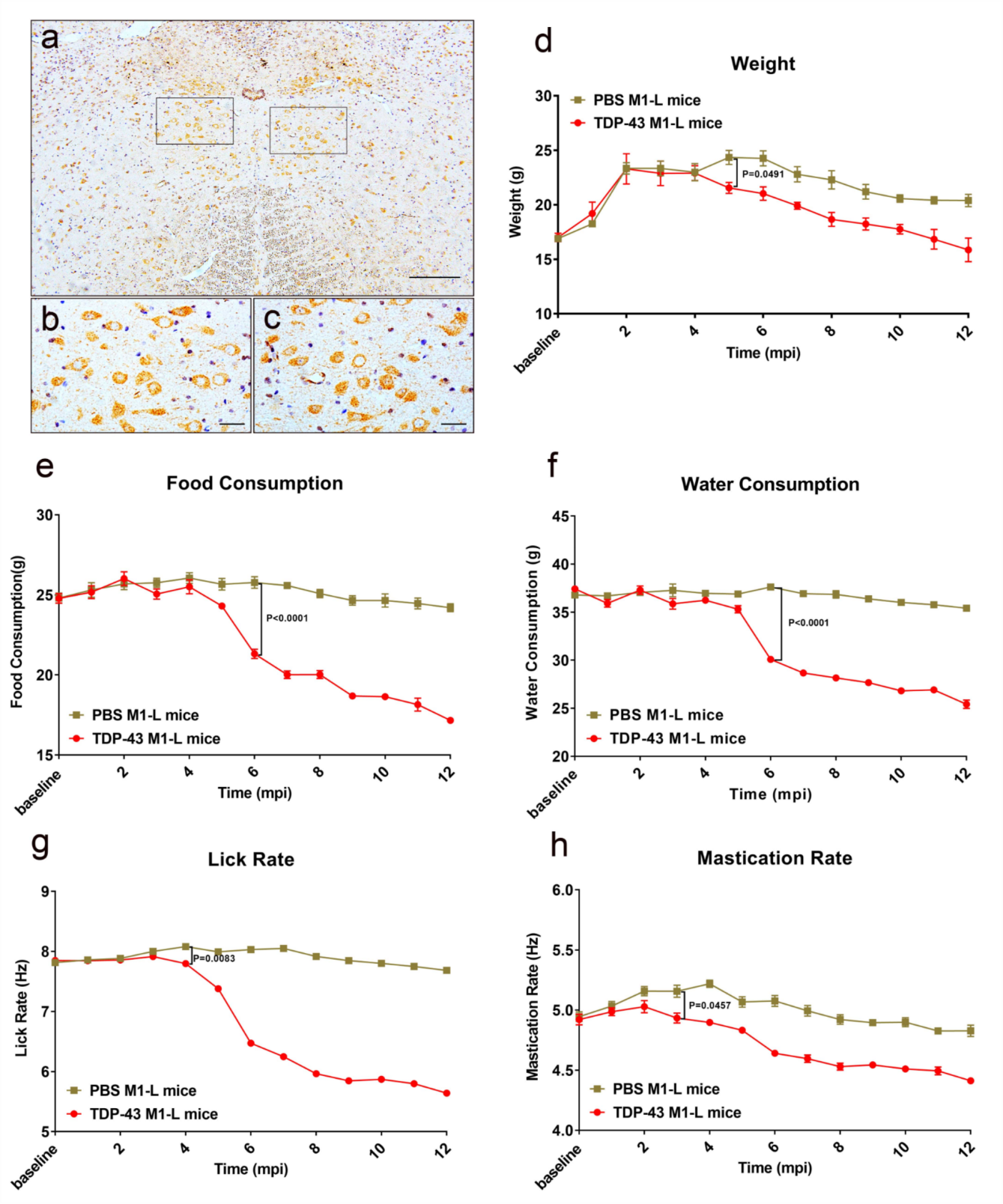
**

**Fig. S5. TDP-43 pathology distribution in medulla oblongata and swallowing function analysis of TDP-43 PFFs M1-L mice.** pTDP-43 IHC staining in the medulla oblongata from TDP-43 PFFs M1-L mice at 8 mpi (n = 3 mice/group) in the ipsilateral (Ipsi) and contralateral (Contra) side of injection (**a-c**). p409-410 stainings were mainly detected in the Ipsi hypoglossal nucleus (12N, **b**) and Contra 12N (**c**). Indirect assessments of dysphagia were performed by calculation of body weight (**d**) and food consumption (**e**) and water consumption (**f**). n = 9 mice/group. Direct measures of dysphagia were carried out by lick rates (**g**) and mastication rates (**h**). n = 9 mice/group. The error bar in all panels represents the Standard Error of Mean (SEM). Data are the means ± SEM. Statistical analysis was performed using the Student’s t test.


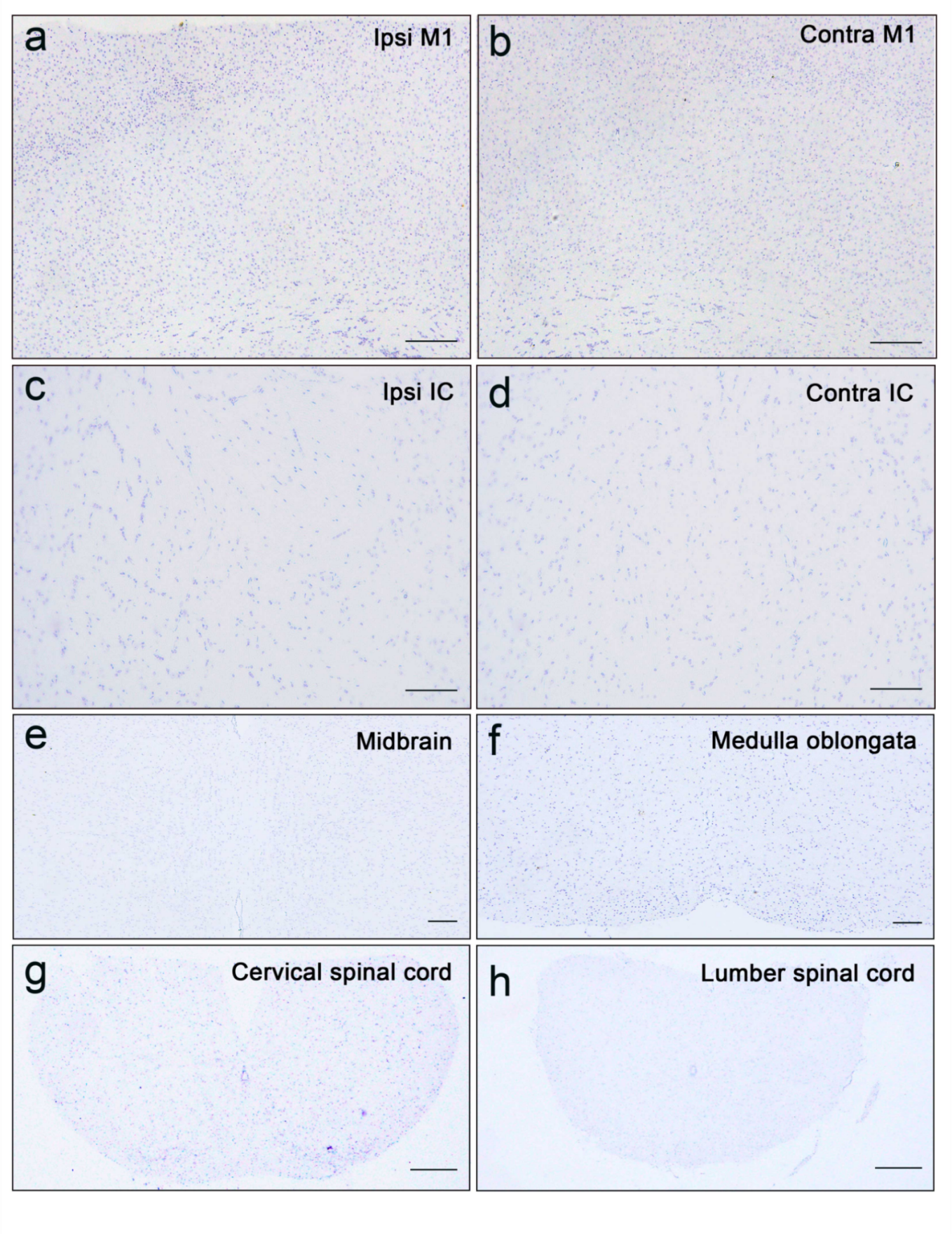


**Fig. S6.** **Lacking of pTDP-43 pathology in CNS segments of TDP-43 PFFs M1-C C57BL/6J mice.** Representative immunohistochemical results displaying the lacking of pTDP-43 pathology in the cortex **(a-b**), IC **(c-d**), midbrain **(e)**, medulla oblongata (**f**), C **(g)** and L **(h)** from TDP-43 PFFs M1-C C57BL/6J mice analyzed at 20 mpi (n = 3 mice/group) in the ipsilateral and contralateral side of injection.


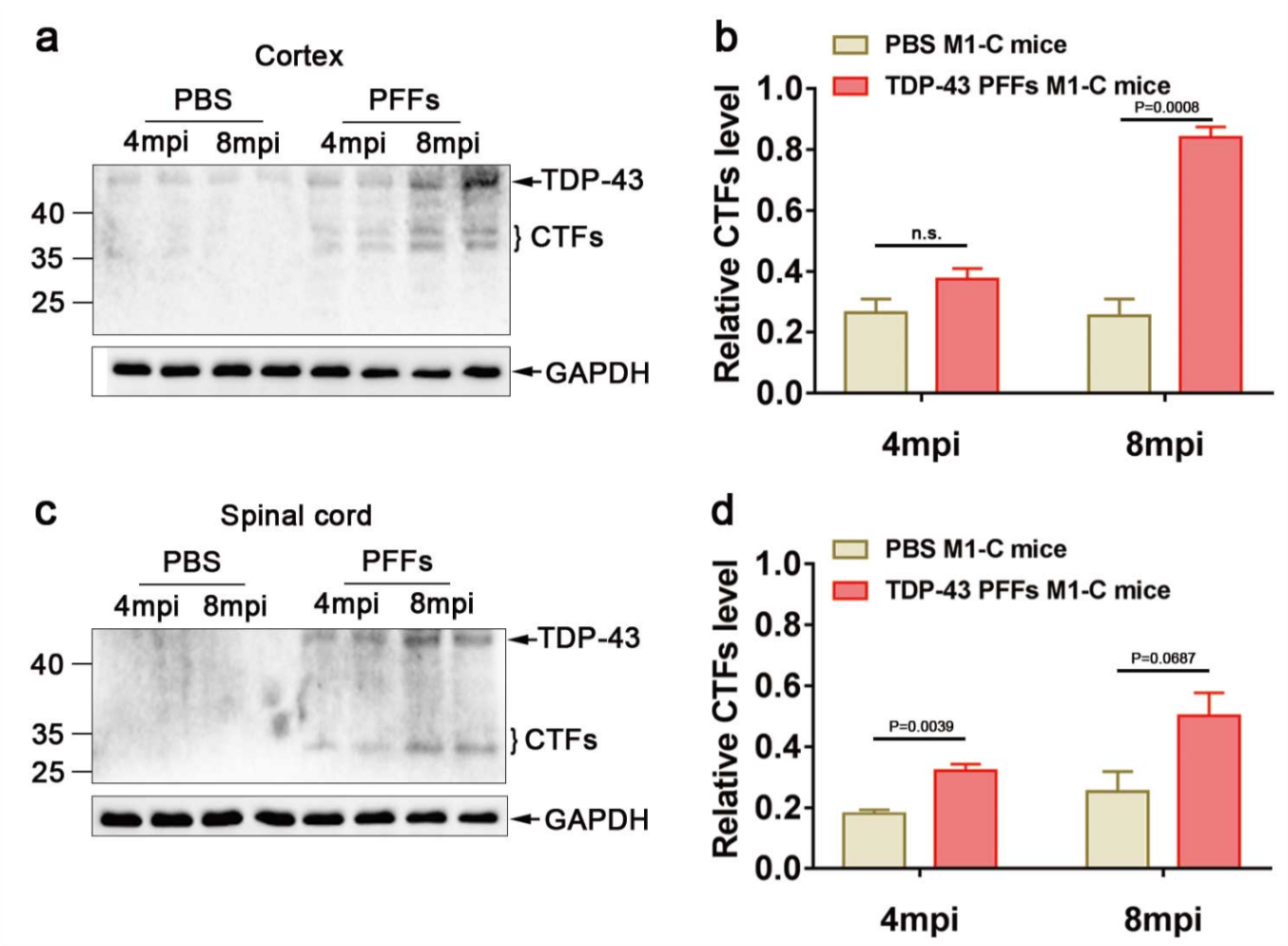


**Fig. S7. The expression level of C-terminal TDP-43 between TDP-43 PFFs M1-C mice and PBS M1-C mice.** Representative WB images of C-terminal TDP-43 in the insoluble fraction of cortex **(a)** and cervical spinal cord **(c)** of TDP-43 PFFs M1-C mice and PBS M1-C mice at different time point and quantification **(b, d).** Blots were probed for GAPDH as a loading control (Bottom). n=3 mice/age/group. Data are the means ± SEM. Statistical significance was analyzed using the Student’s t test and Mann-Whitney test.


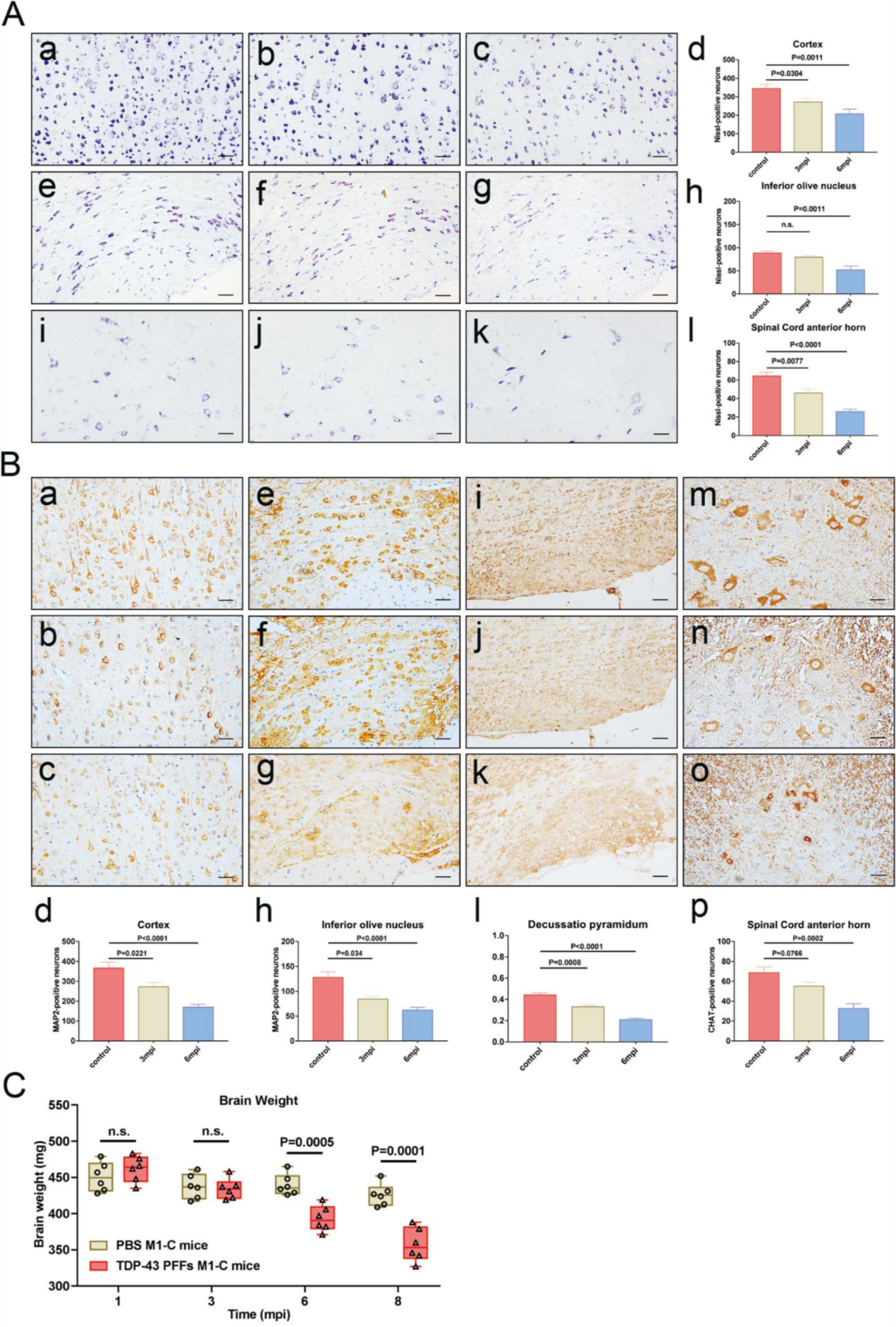


**Fig. S8. Immunohistochemistry and Nissl Staining in different brain regions of TDP-43 PFFs M1-C mice and PBS M1-C mice and the analysis of brain weight**. **A. (a-d)** Representative images of Nissl-positive neurons in M1 of PBS M1-C mice at 6 mpi **(a)**, TDP-43 PFFs M1-C mice at 3 mpi **(b)**, 6 mpi **(c)** and stereology quantification **(d)**. **(e-h)** Representative images of Nissl-positive neurons in Inferior Olivary Nucleus (ION) of PBS M1-C mice at 6 mpi **(e)**, TDP-43 PFFs M1-C mice at 3 mpi **(f)**, 6 mpi **(g)** and stereology quantification **(h)**. **(i-l)** Representative images of Nissl-positive neurons in Cs of PBS M1-C mice **(i)** at 6 mpi, TDP-43 PFFs M1-C mice at 3 mpi **(j)**, 6 mpi **(k)** and stereology quantification **(l)**. n = 3 mice/group. Scale bar, 50 µm. **B. (a-d)** Representative images of MAP2-positive neurons in M1 of PBS M1-C mice at 6 mpi **(a)**, TDP-43 PFFs M1-C mice at 3 mpi **(b)**, 6 mpi **(c)** and stereology quantification **(d)**. **(e-h)** Representative images of MAP2-positive neurons in ION of PBS M1-C mice at 6 mpi **(e)**, TDP-43 PFFs M1-C mice at 3 mpi **(f)**, 6 mpi **(g)** and stereology quantification **(h)**. **(i-l)** Representative images of NF-positive nerve fibers in py of PBS M1-C mice at 6 mpi **(i)**, TDP-43 PFFs M1-C mice at 3 mpi **(j)**, 6 mpi **(k)** and stereology quantification **(l)**. **(m-p)** Representative images of CHAT-positive neurons in Cs of PBS M1-C mice at 6 mpi **(m)**, TDP-43 PFFs M1-C mice at 3 mpi **(n)**, 6 mpi **(o)** and stereology quantification **(p)**. n = 3 mice/group. Scale bar, 50 µm. **C.** Brain weight of TDP-43 PFFs M1-C mice and PBS M1-C mice at 1, 3, 6, 8 mpi. n = 6 mice/group.


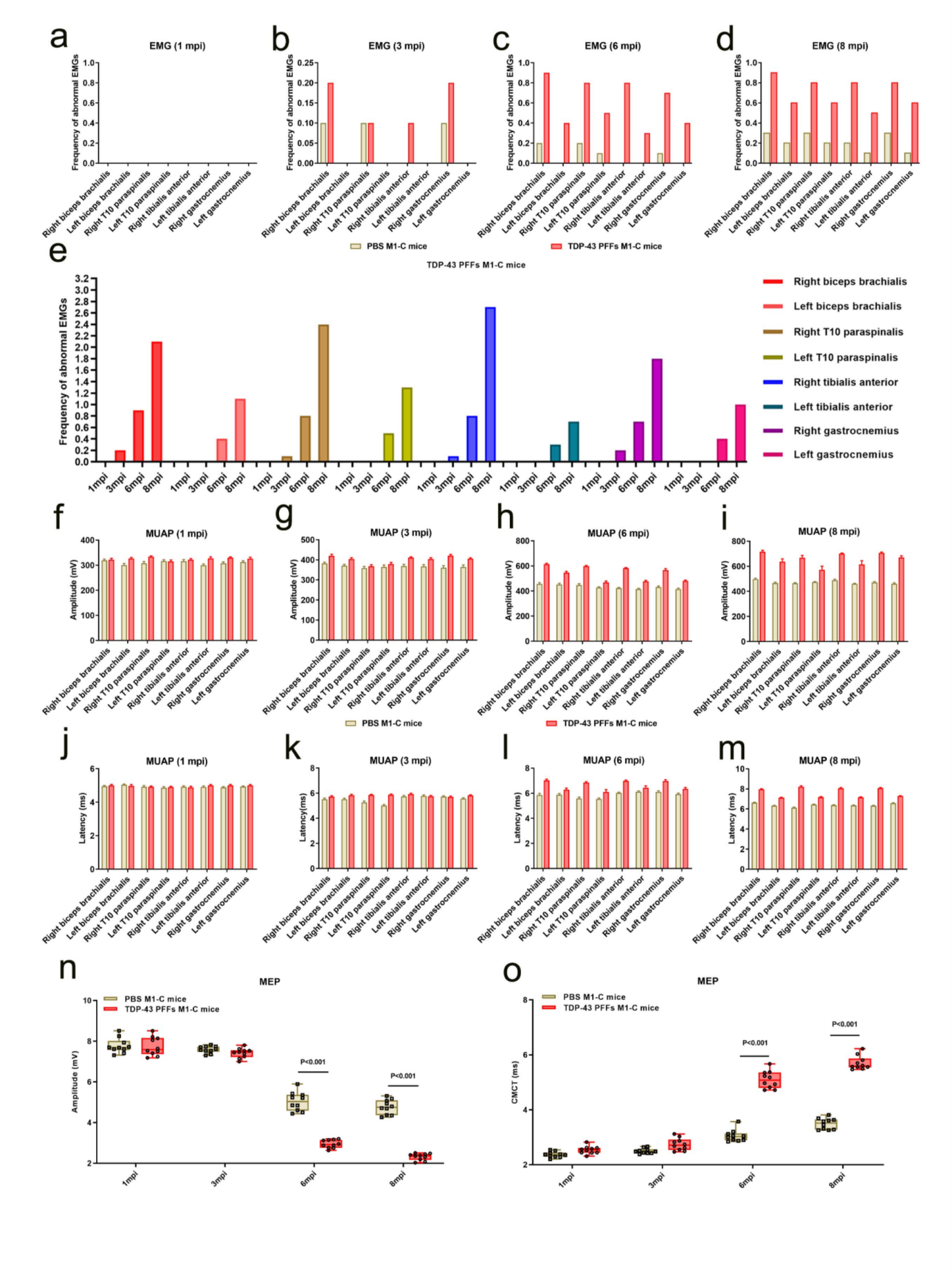


**Fig. S9. Neurophysiology of TDP-43 PFFs M1-C mice and PBS M1-C mice**. The frequency of abnormal spontaneous activity in bilateral biceps brachialis, T10 paraspinals, tibialis anterior, and gastrocnemius muscles of TDP-43 PFFs M1-C mice and PBS M1-C mice at 1 mpi **(a)**, 3 mpi **(b)**, 6 mpi **(c)**, and 8 mpi **(d)**. The frequency of abnormal spontaneous activity in TDP-43 PFFs M1-C mice was developed in a time-dependent manner **(e)**. The amplitude of MUAPs at 1 mpi **(f)**, 3 mpi **(g)**, 6 mpi (h), 8 mpi (i) and latency of MUAPs at 1 mpi **(j)**, 3 mpi **(k****)**, 6 mpi **(l)**, 8 mpi **(m)** of TDP-43 PFFs M1-C mice and PBS M1-C mice were analyzed. Amplitude **(n)** and CMCT **(o)** of L-cMEP of TDP-43 PFFs M1-C mice and PBS M1-C mice at 1 mpi, 3 mpi, 6 mpi and 8 mpi. n = 10 mice/group. The error bar in all panels represents the Standard Error of Mean (SEM). Data are the means ± SEM. Statistical analysis was performed using the Student’s t test.

**
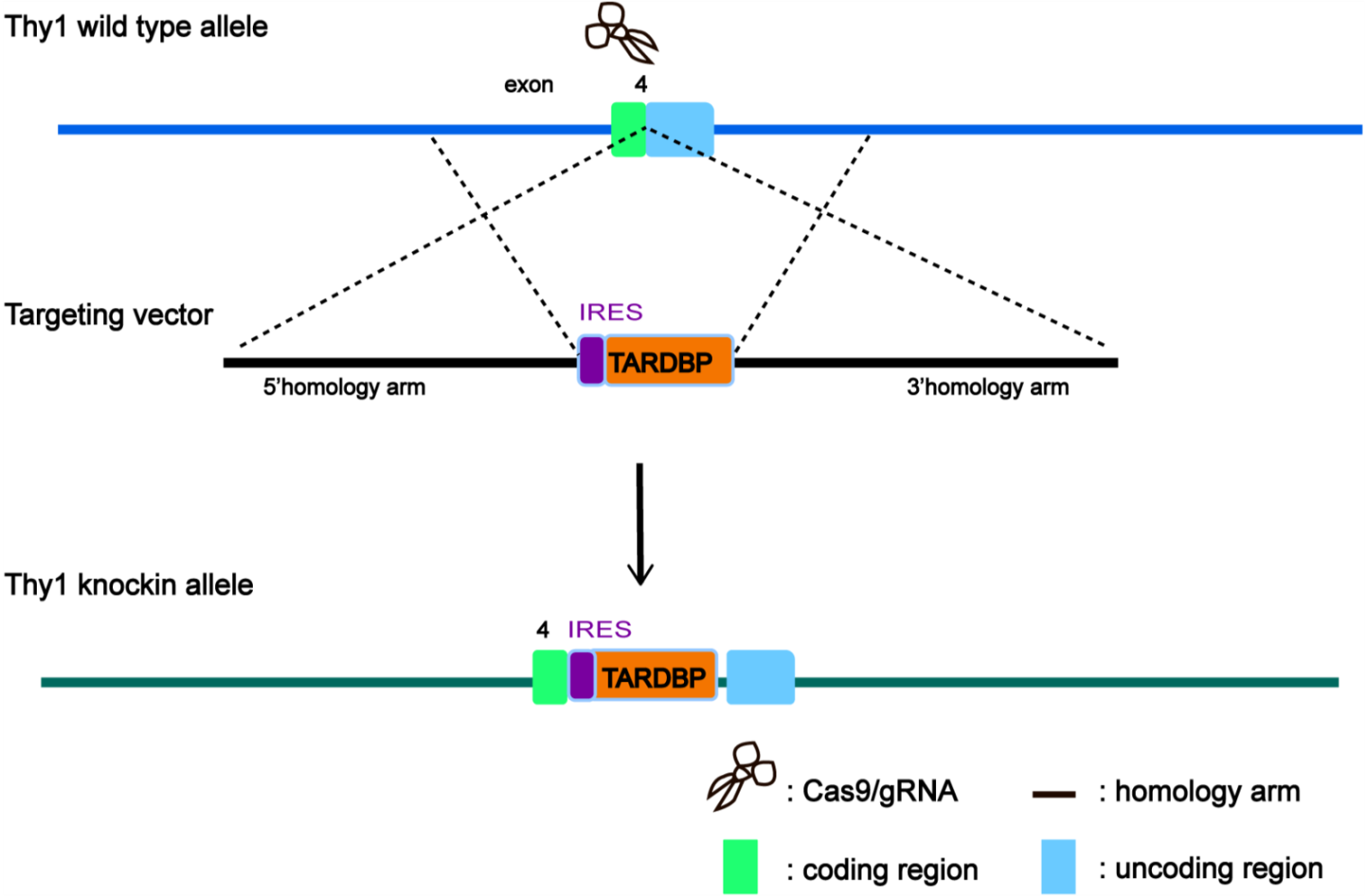
**

**Fig. S10. IRES-TARDBP gene knocked into the stop exon of the Thy1 gene via CRISPR/Cas9 strategy.**

**Supplementary Tables**

**Supplementary Table 1. Primers used to identify the genotype of Thy1-e(IRES-TARDBP) 1 mice**

| Primers | Sequence (5’→3’) |
| --- | --- |
| P1 | CTCCCGGCACCCCTTCTCTATCC |
| P2 | CCTCCTACCCCTTTCCCCATCATT |
| P3 | CCTCCCGGCACCCCTTCTCTATCC |
| P4 | TGGGGTACCTTCTGGGCATCCTTC |

**Supplementary Table 2.** **Antibodies used in the study**

| **Name antibody** | **Epitope** | **Source (Cat. #)** | **Host** | **Method**  **(dilution or concentration)** | | |
| --- | --- | --- | --- | --- | --- | --- |
|  |  |  |  | IHC | IF | WB |
| Anti TAR DNA-Binding Protein 43 (TDP-43), phospho Ser409/410 mAb | pTDP-43 (phosphorylated at Ser409/Ser410) | Cosmo Bio Co., Ltd (TIP-PTD-M01) | Mouse |  |  | 1:600 |
| Phospho-TDP43 (Ser409/410) Polyclonal Antibody | pTDP-43 (phosphorylated at Ser409/Ser410) | Proteintech (22309-1-AP) | Rabbit |  | 1: 300 |  |
| Purified anti-TDP43 Phospho (Ser409/410) Antibody | pTDP-43 (phosphorylated at Ser409/Ser410) | Biolegend (829901) | Rat | 1: 200 | 1: 300 |  |
| TDP-43 (human specific) Monoclonal antibody | Human TDP-43 | Proteintech (60019-2-Ig) | Mouse | 1:500 |  | 1:2000 |
| Tardbp Rabbit Polyclonal Antibody | Mouse TDP-43 | ORIGENE (AP55314SU-N) | Rabbit |  |  | 1:500 |
| TDP43 (D9R3L) Rabbit mAb | TDP-43 | Cell Signaling (89789) | Rabbit |  |  | 1:2000 |
| Anti-TDP-43(C-terminal) antibody | TDP-43(C-terminal) | Millipore (T1580) | Rabbit |  |  | 1:2000 |
| Anti-Vesicular Acetylcholine Transporter (VAChT) Antibody | ChAT | Millipore (ABN100) | Goat | 1:100 |  |  |
| Anti GFAP antibody | GFAP | Abcam (ab4674) | Chicken |  | 1: 600 |  |
| Anti Iba1 antibody | Iba1 | Wako (019-19741) | Rabbit |  | 1: 100 |  |
| Anti-Myelin Basic protein antibody | MBP | Abcam (ab40390) | Rabbit |  | 1: 200 |  |
| Anti-Neurofilament heavy polypeptide antibody | NF | Abcam (ab7795) | Mouse | 1:100 | 1: 100 |  |
| Ubiquitin Polyclonal antibody | ubiquitin | Proteintech (10201-2-AP) | Rabbit |  | 1: 100 |  |
| Anti MAP-2 antibody | MAP-2 | Abcam (ab5392) | Chicken | 1:200 | 1: 200 |  |
| Rhodamine Red™-X (RRX) AffiniPure Donkey Anti-Mouse IgG (H+L) |  | Jackson ImmunoResearch (715-295-151) | Donkey |  | 1: 600 |  |
| Cy™3 AffiniPure Donkey Anti-Rabbit IgG (H+L) |  | Jackson ImmunoResearch (711-165-152) | Donkey |  | 1: 600 |  |
| Cy™2 AffiniPure Donkey Anti-Rabbit IgG (H+L) |  | Jackson ImmunoResearch (711-225-152) | Donkey |  | 1: 800 |  |
| Cy™2 AffiniPure Donkey Anti-Chicken IgG (H+L) |  | Jackson ImmunoResearch (703-225-155) | Donkey |  | 1: 800 |  |
| Cy™2 AffiniPure Donkey Anti-Goat IgG (H+L) |  | Jackson ImmunoResearch (705-225-147) | Donkey |  | 1:800 |  |
| Anti-Mouse IgG (H+L) HRP Conjugate |  | Promega (W4021) | Goat |  |  | 1: 2500 |
| GAPDH |  | Abcam (ab8245) | Mouse |  |  | 1: 1000 |
